# Motor defects in a *Drosophila* model for spinal muscular atrophy result from SMN depletion during early neurogenesis

**DOI:** 10.1371/journal.pgen.1010325

**Published:** 2022-07-25

**Authors:** Stuart J. Grice, Ji-Long Liu

**Affiliations:** 1 Medical Research Council Functional Genomics Unit, Department of Physiology, Anatomy and Genetics, University of Oxford, Oxford, United Kingdom; 2 School of Life Science and Technology, Shanghai, Tech University, Shanghai, China; The Jackson Laboratory, UNITED STATES

## Abstract

Spinal muscular atrophy (SMA) is the most common autosomal recessive neurodegenerative disease, and is characterised by spinal motor neuron loss, impaired motor function and, often, premature death. Mutations and deletions in the widely expressed *survival motor neuron 1* (*SMN1*) gene cause SMA; however, the mechanisms underlying the selectivity of motor neuron degeneration are not well understood. Although SMA is degenerative in nature, SMN function during embryonic and early postnatal development appears to be essential for motor neuron survival in animal models and humans. Notwithstanding, how developmental defects contribute to the subversion of postnatal and adult motor function remains elusive. Here, in a *Drosophila* SMA model, we show that neurodevelopmental defects precede gross locomotor dysfunction in larvae. Furthermore, to specifically address the relevance of SMN during neurogenesis and in neurogenic cell types, we show that SMN knockdown using neuroblast-specific and pan-neuronal drivers, but not differentiated neuron or glial cell drivers, impairs adult motor function. Using targeted knockdown, we further restricted SMN manipulation in neuroblasts to a defined time window. Our aim was to express specifically in the neuronal progenitor cell types that have not formed synapses, and thus a time that precedes neuromuscular junction formation and maturation. By restoring SMN levels in these distinct neuronal population, we partially rescue the larval locomotor defects of *Smn* mutants. Finally, combinatorial SMN knockdown in immature and mature neurons synergistically enhances the locomotor and survival phenotypes. Our in-vivo study is the first to directly rescue the motor defects of an SMA model by expressing *Smn* in an identifiable population of *Drosophila* neuroblasts and developing neurons, highlighting that neuronal sensitivity to SMN loss may arise before synapse establishment and nerve cell maturation.

## Author summary

Spinal muscular atrophy (SMA) is the most common genetic cause of infant mortality and leads to the degeneration of the nerves that control muscle function. Loss-of-function mutations in the widely expressed *survival motor neuron 1* (*SMN1*) gene cause SMA, but how low levels of SMN protein cause the neuronal dysfunction is not known. Although SMA is a disease of nerve degeneration, SMN function during nerve cell development may be important, particularly in severe forms of SMA. Nevertheless, how the defects during development and throughout early life contribute to the disease is not well understood. We have previously demonstrated that SMN protein becomes enriched in neuroblasts, which are the cells that divide to produce neurons. In the present study, motor defects observed in our fly model for SMA could be rescued by restoring SMN in neuroblasts alone. In addition, we show that knocking down SMN in healthy flies within the same cell type causes impaired motor function. The present study shows that the manipulation of SMN in a developmentally important cell type can cause motor defects, indicating that a period of abnormal neurodevelopment may contribute to SMA.

## Introduction

Survival motor neuron (SMN) is an essential protein that functions in the biogenesis of spliceosomal small nuclear ribonucleoproteins (snRNPs), which subsequently mediate pre-mRNA splicing [1]. Loss-of-function mutations in the *SMN1* gene cause the disease spinal muscular atrophy (SMA), which is characterised by the selective loss of alpha motor neurons of the spinal cord, muscle wasting and, in most severe cases, premature death in infancy [2].

Since the identification of the disease-associated gene *SMN1* in 1995 [2], the drive to uncover the mechanisms underlying SMA pathogenesis has been complicated by the pleiotropic nature of the *SMN* locus [3], coupled with the varied levels of SMN protein in human and animal models [4–6]. There has been considerable debate about how aberrations in both the canonical and non-canonical motor neuron-specific functions of SMN may lead to the observed motor neuron selectivity [7]. SMN has been shown to play a fundamental role in snRNP and messenger ribonucleoprotein (mRNP) biogenesis [8], whilst also being involved in mRNA trafficking and local translation, cytoskeletal dynamics, endocytosis and ubiquitin homeostasis (reviewed in [3,9]). In addition, the nature of SMA pathology, and of the animal models engineered to study the disease, are greatly affected by the systemic, temporal and spatial levels of SMN protein [5,6]. In humans, in addition to *SMN1*, SMN is also encoded by a second paralogous gene called *SMN2*, which, owing to a mutation affecting exon 7 splicing, generates comparatively low levels of full-length SMN protein [10,11]. Due to the fact that *SMN2* copy number can also vary between individuals, there is a broad spectrum of disease severity that, at the population level, correlates with the amount of *SMN2*-derived wild-type SMN protein [4]. As SMN levels decrease, disease severity increases, the motor defects become more pronounced, and many more cell and tissue types present with phenotypes caused by loss of the protein [12,13].

Classed as a ubiquitous protein, SMN localises to the cytoplasm and nucleus, and can be observed in many RNP-enriched subcellular foci, such as Gems [14], nucleoli [15], U bodies [16] and Cajal bodies [14]. Cells do not necessarily require organised Gems, U Bodies and Cajal bodies to survive; however, evidence shows that these celluar foci promote the efficient clustering of the RNA processing factors required in embryonic, dividing and metabolically active cells [17]. SMN protein level and associated snRNP assembly are highest during embryonic development, and are substantially downregulated postnatally [18] and as cells differentiate and mature [19–21]. Furthermore, severe SMN loss can lead to developmental defects, with a hierarchy of cell types, many of which are uncharacterised, having differing sensitivities to a reduction in the level of the protein [6,12].

Undoubtedly, the alpha motor neurons are particularly sensitive to SMN reduction. Notwithstanding, it is not known how this selectivity manifests in its entirety (i.e., whether it is a result of aberrations in set-up or degeneration, or if it is through a non-cell autonomous mechanism). Previous research has shown that the selective loss of SMN in motoneuronal progenitors is sufficient to cause SMA like phenotypes [22]. Furthermore, restoration of SMN in mature motoneurons only rescued the SMA phenotype partially [23,24], whereas motor neuron-specific SMN reduction in wild type mice fails to recapitulate the entirety of the disease phenotypes, highlighting the importance of neuronal development. Motor neuron loss is also a relatively late feature in SMN patients and mammalian models [25, 26], although patients with type 1 SMA present with neuromuscular junction (NMJ) maturation defects during fetal development [27]. Importantly, when performing rescue studies using mouse and *Drosophila* SMA models, early stage ubiquitous restoration of SMN results in the greatest improvement in motor function and animal survival [26,28,29]. This is supported by evidence from patient clinical trials [30] and early versus later treatment of SMA mice [6,31–37]. To complement these findings, SMN reduction in young adult mice caused more modest phenotypes when compared with mice in which SMN was knocked down at an earlier developmental time point [6, 26,28]. Furthermore, *Drosophila* studies using *Smn* mutant models have reported severe growth defects and considerable developmental retardation, in addition to motor and NMJ dysfunction [5,21,38–41]. In mouse embryos, although no overt developmental outgrowth defects have been observed [42], defective radial outgrowth and poor Schwann ensheathment led to some axons degenerating postnatally [43]. Altogether, this research highlights that the cause of SMA may not be solely through classical neurodegenerative processes, but via a combinatory multi-cell type mechanism that may be sensitised by neurodevelopmental abnormalities. An understanding of the precise nature of the developmental requirements of SMN, and how perturbations in SMN protein level leads to defects that manifest in progenitor and non-differentiated neuronal cell types, is important for SMA treatment.

The aim of the present study was to understand how manipulation of SMN protein level during specific periods of neurogenesis can cause and modify the phenotypes present in *Drosophila* models for SMA. The aim was to restrict SMN manipulation to the neuronal progenitor cell types that have not yet formed synapses, and to a period that precedes NMJ maturation. To achieve this, knockdown and rescue studies were used during the waves of proliferation and differentiation in the larval and pupal central nervous system (CNS). The classical GAL4 and the more targeted GAL80 repression systems were used to allow for spatiotemporal transgene expression [44]. The reduction of SMN in neuroblasts and undifferentiated neurons, but not subsequently in differentiated subpopulations of neurons, caused motor defects. In the reciprocal experiment, neurodevelopmental and motor phenotypes are partially rescued by expressing SMN in neuroblasts and immature neurons. Finally, combinatorial SMN knockdown was carried out in immature and mature neurons, which synergistically enhanced the locomotor and survival phenotypes in the present model. This in-vivo study contributes to the understanding of how developmental abnormalities can contribute to the motor defects synonymous with the pathology of SMA. Furthermore, by selectively manipulating SMN in an identifiable population of neuroblasts and developing neurons, we highlight the importance of SMN in *Drosophila* neurodevelopment.

## Results

### Developmental arrest precedes larval locomotion dysfunction in Smn mutants

**Fig 1:**
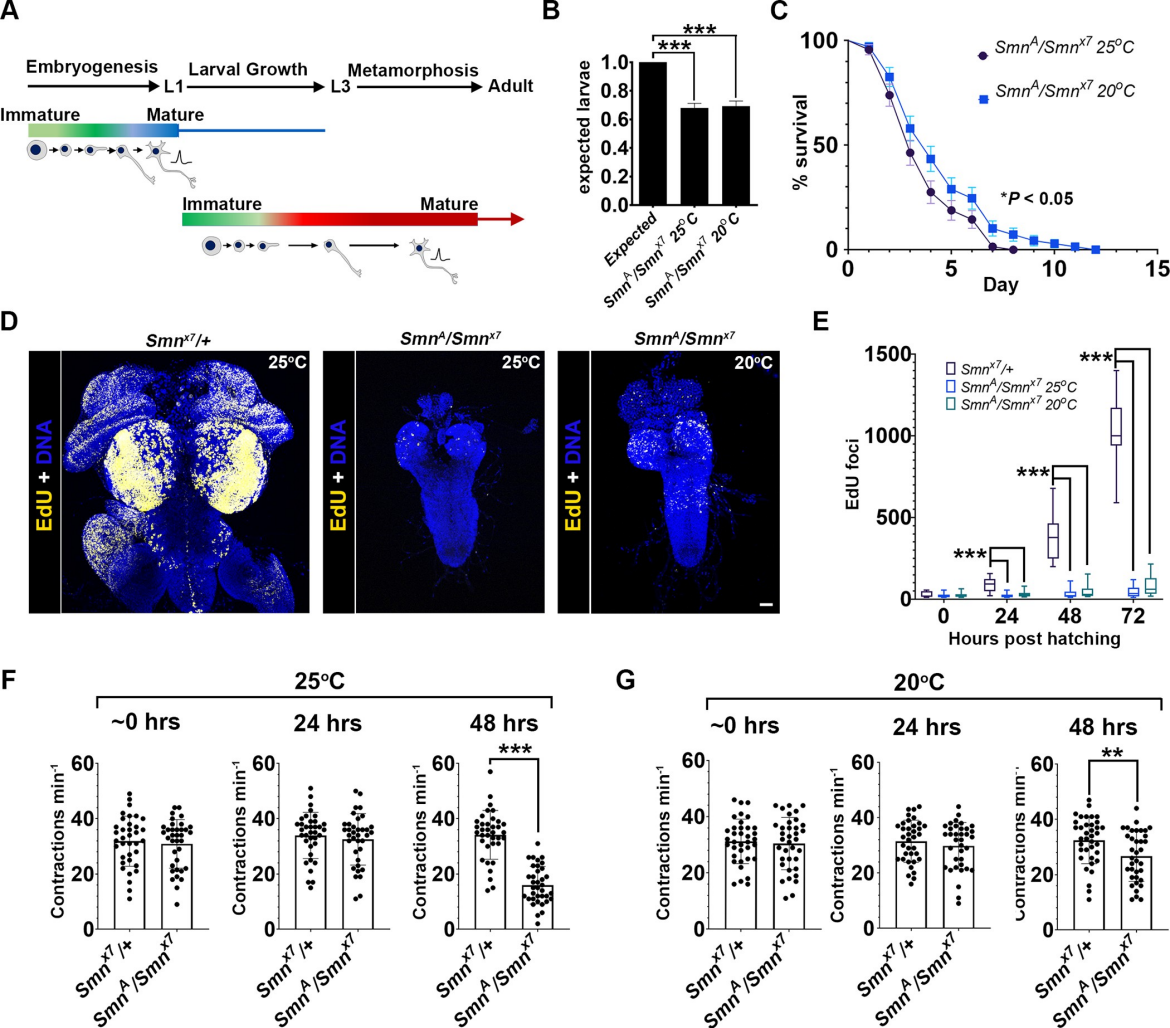
Neurodevelopmental defects precede gross locomotor dysfunction in *Smn* mutants. (A) The *Drosophila* developmental stages. *Drosophila* go through two major rounds of neurogenesis during embryogenesis, in which the larval nervous system is set up, and during the larval stage, in which the adult neurons and glia are formed (these mature during metamorphosis and early adult life); (B) *Smn*^*x7*^*/Smn*^*A*^ embryos were collected and the number of hatched larvae scored for each genotype and normalised to numbers observed from control *Smn*^*x7*^*/+*. Fewer *Smn*^*x7*^*/Smn*^*A*^ larvae hatched from embryos at 25°C and 20°C (error bars [SEM] represent three experiments, each with *n* > 90; ***P < 0.001; Kruskal–Wallis test with Dunn’s multiple comparisons); (C) *Smn*^*x7*^*/Smn*^*A*^ larvae survived for a median of 3 and 4 days in a developmentally immature state when kept at 25°C and 20°C, respectively (error bars [SEM] represent three experiments, each with *n* > 60, *P < 0.05 Mantel-Cox). (D) confocal images of 5-ethynyl-2’-deoxyuridine (EdU) incorporation in *Smn*^*x7*^*/+* and *smn*^*x7*^*/smn*^*A*^ trans-heterozygous larvae aged 5 days; (E) counts of EdU-containing foci in the thoracic ganglion over 72 h for larvae kept at 25°C and 20°C. At both temperatures, *Smn*^*x7*^*/Smn*^*A*^ failed to show an increase in EdU incorporation. (**P < 0.01; ***P < 0.001, *n* = 15 per genotype; Kruskal–Wallis test with Dunn’s multiple comparisons); (F and G) body wall contractions were scored at 0, 24 and 48 h after hatching over a 1-min period at (F) 25°C and (G) 20°C. *Smn*^*x7*^/*Smn*^*A*^ larvae underwent significant contraction defects at 48 h at both 25°C (***P < 0.001; Kruskal–Wallis test *n* = 36) and 20°C (**P < 0.01; Kruskal–Wallis test *n* = 36). All error bars [SEM]Scale bar = 20 μm.

During *Drosophila* embryonic neurogenesis, the nervous system required for larval life is generated (Fig 1A) [45–47]. Larvae then develop through three instar stages (L1–L3) before they pupate and become adults. During larval life, a second wave of neurogenesis occurs, and about 90% of adult neurons are created [48]. Loss-of-function and null *Drosophila Smn* mutants survive until larval stage on a maternal contribution of SMN protein [40]. This shortened lifespan is observed in the micro-deletion S*mn^x7^*, which removes the *Smn* coding region and is therefore classified as a null [39], and the point mutant *Smn^A^*, which acts as a loss-of-function [40]. In the present study, *Smn^A^*/*Smn^x7^* trans-heterozygotes (herein referred to as *Smn* mutants) were used to reduce the influence of genetic background. Although no embryonic defects have been described previously [49], significantly fewer *Smn* mutant larvae hatching than expected were observed at 25°C and 20°C (Fig 1B). Both 25°C and 20°C are the commonly used rearing temperatures that confer approximately an 11-day and 14-day lifecycle in the fly, respectively. For these experiments, the use of the life cycle lengths allowed the timeline of the developmental and motor phenotypes observed in the *Smn* mutants to be plotted more accurately. Homozygotes of both *Smn^A^* and *Smn^x7^* can live for several days in a developmentally immature larval state [5,21,39,40], and die as larvae when maternal SMN becomes depleted. The remaining hatched *Smn* mutants were tested and a comparable survival timeline was found, with larvae dying at a median of 3 and 4 days when kept at 25°C and 20°C, respectively (Fig 1C).

At the end of L1 (24 h after hatching, approximately 48 h after egg laying), most neuroblasts exit quiescence and start to divide [48]. As neuroblasts exit quiescence, they become enriched with SMN [21], and clonal knockout of *Smn* in neuroblasts has been shown to limit cell division and alter the clonal structure of the daughter cells generated from the SMN-deficient stem cells [21]. To determine the timing of proliferation defects in relation to *Smn* mutant larval lifespan, the number of nuclei stained positively for s-phase incorporated 5-ethynyl-2’-deoxyuridine (EdU) over a 72-h period were scored. Except for a small population of continually dividing neuroblasts (some lateral and mushroom body neuroblasts bypass quiescence at the end of embryogenesis), low levels of EdU incorporation were observed in *Smn* mutants. A significant difference in EdU foci can be observed from 24 h when reared at both 25°C and 20°C (Fig 1D and 1E). This difference widened at 48 and 72 h after hatching (Fig 1E). After 72 h as larvae, the CNS of the remaining *Smn* mutant larvae remained significantly under-developed compared with wild-type larvae, when cultured at 25°C and 20°C (Fig 1D), highlighting that neuroblasts fail to significantly proliferate during the attenuated larval survival period. These data suggest that neuroblasts either fail to reactivate or that they generate only a nominal number of immature neurons and glia.

As SMA is a disorder of the motor system, larval locomotor dysfunction is often used as a proxy for motor abnormalities. The number of larval peristaltic muscle contractions that drive larval movement was next quantified. This is a method that has previously been used in the analysis of *Drosophila* models of neuropathy and SMA [50,51]. These contractions involve the rhythmic and sequential contraction of body-wall muscles, and are controlled by an intricate circuit of motor neurons and excitatory and inhibitory interneurons [52]. Contractions were scored at 0, 24 and 48 h after hatching over a 1-min period. Previous studies have identified that motor function defects become apparent after approximately 3 days in *Smn* mutant larvae [39,40]. At time point 0 and 24, both control and *Smn* mutants undergo a comparable number of contractions at 25°C and 20°C (Fig 1F and 1G). At larval age 48 h, the number of Smn mutant larvae contractions reduced by approximately 53% at 25°C (Fig 1F) and 18% at 20°C (Fig 1G), when compared to controls. Additionally, larvae were filmed for 1 min and tracked the distance travelled on an agar surface. Again, at 48 h, larvae travelled significantly less distance compared to controls (S1 Fig). At this time point, however, *Smn* mutant larvae are significantly smaller than wildtypes and do not represent the same instar based on size or morphology. In summary, gross developmental defects, which include the pausing of neurogenesis, precede movement dysfunction in the larval SMA model.

### SMN knockdown in neurogenic cell types cause larval and adult locomotor dysfunction

**Fig 2:**
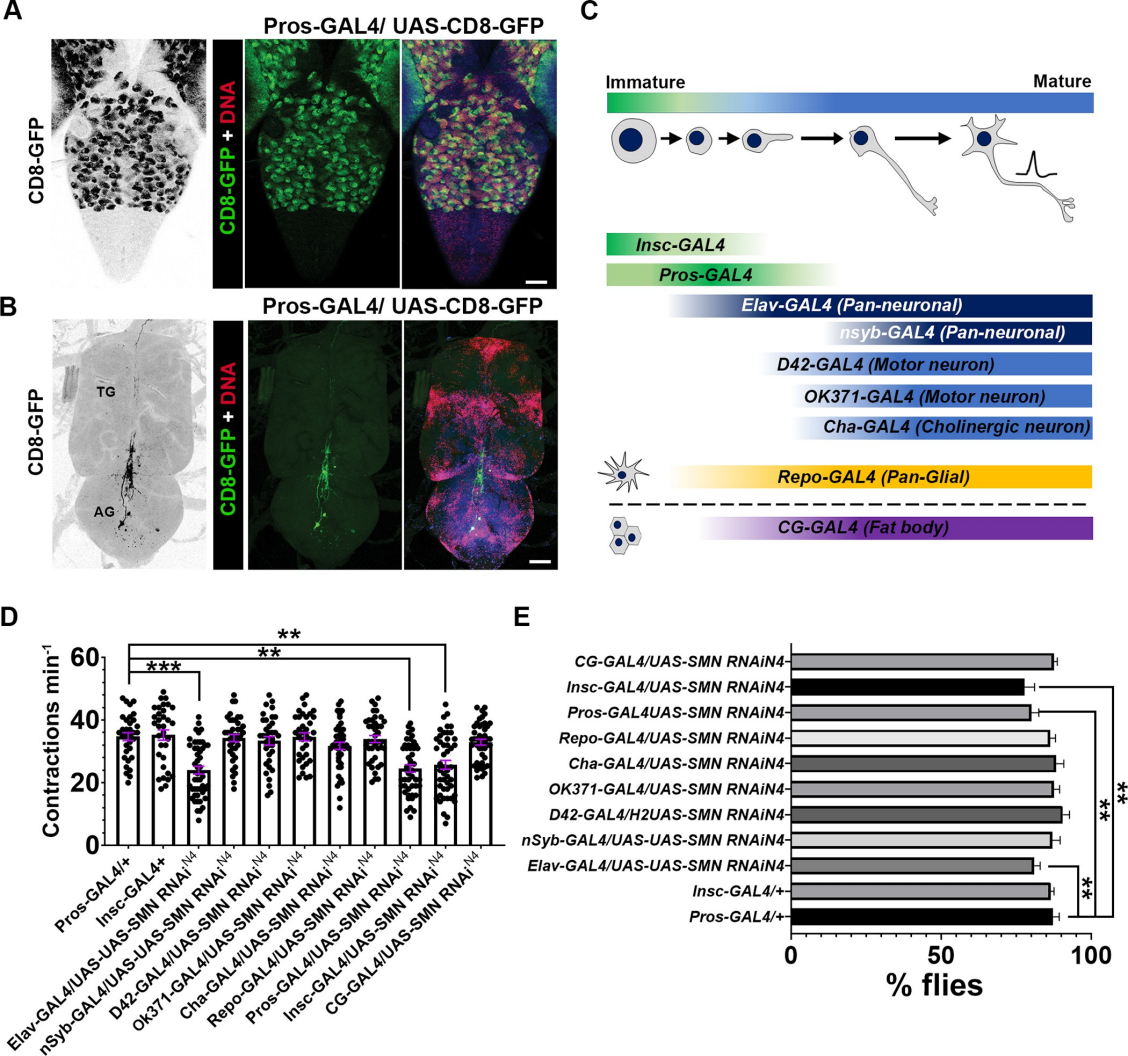
*Survival motor neuron* (*Smn*) knockdown in neurogenic cell types leads to larval developmental defects and locomotor dysfunction. (A) *Pros-GAL4* driven expression of membrane-bound CD8-green fluorescent protein (GFP) in the larval central nervous system. GFP expression is observed in the post-embryonic neuroblasts and their immature daughter cells; (B) *Pros-GAL4* expression in the adult ventral nerve cord. No neurons within the thoracic ganglion show visible expression. Only a small number of neurons, which reside in the abdominal ganglion within the ventral nerve cord of the adult, expressed *Pros-GAL4*; (C) *GAL4* nervous system expression patterns detailing the neuronal and glial cell type expression patterns; (D–E) SMN was knocked-down (*UAS-SMN-RNAi*^*N4*^) pan neuronally (Elav-GAL4 and nSyb-GAL4) predominantly in motor neurons (*D42-GAL4* and *OK371-GAL4*), cholinergic neurons (*Cha-GAL4*), neuroblasts and undifferentiated daughter cells (*Pros-GAL4 and Insc-GAL4*), pan-glia (*Repo-GAL4*) and in the larval fat body (*CG-GAL4*); (D) body wall contractions were scored at 48 h, with significant differences observed with *Elav-GAL4*, *Pros-GAL4* and *Insc-GAL4* driven *UAS-SMN-RNAi*^*N4*^ (**P < 0.01, ***P < 0.00, Kruskal–Wallis test with Dunn’s multiple Comparisons; *n* > 20); (E) day of pupariation formation (three experiments each with *n* > 50; Kruskal–Wallis test with Dunn’s multiple comparisons) data showing that Insc-GAL4 and Pros-GAL4 SMN knockdown leads to a delay in time to pupariation; All error bars [SEM]. Scale bar = 10 μm.

The coincidence of locomotor and developmental defects, and the ever-depleting SMN levels in *Drosophila Smn* mutants, make it difficult to identify the cellular mechanisms leading to the deterioration of neuronal function. With our interest in immature neuronal identities, the aim was to knockdown SMN by limiting *Smn* RNAi expression to neuroblasts and their daughter cells and to compare the results with pan-neuronal, neuronal subtype, glial cell and fat body *Smn* RNAi expression. Specifically, *Smn* knockdown was carried out using a RNAi hairpin construct *SMN-RNAi^N4^*. When expressed ubiquitously, *SMN-RNAi^N4^* presents as a hypomorph, with most flies dying at early pupal stage [39]. For expression in neuroblasts, the previously reported Inscutable-GAL4 (Insc-GAL4) driver was used [53,54]. Insc-GAL4 is expressed in most embryonic and larval neuroblasts and their immature progeny [53], with a pattern of expression (S2A and S2B Fig) analogous to the enrichment of SMN observed in the post-embryonic neuroblasts [21] (S2C Fig). Expression of *SMN-RNAi^N4^* removes the enrichment of SMN in these cells, although SMN is still present at low levels (S2D Fig). In addition, a driver containing a Prospero (Pros) regulatory sequence (P[pros-GAL4.U]) (Pros-GAL4) was acquired. Prospero protein drives the expression of neural differentiation genes and represses neuroblast stem cell identity and cell cycle proliferation genes. Prospero mRNA is expressed in the neuroblast, and the protein is asymmetrically localized to the neuroblast basal cortex during division, resulting in its partitioning into the daughter cells [55,56]. It was, therefore, speculated that the driver would express in neuroblasts and their daughter cells and, thus, provide an intermediary between Insc-Gal4 and drivers expressing pan-neuronally in differentiated neurons. To characterise the pattern of Prospero-GAL4 expression, UAS-CD8-GFP was driven using Pros-GAL4 and green fluorescent protein (GFP) localisation was noted. GFP was expressed in neuroblasts and daughter cells in the larval CNS (Fig 2A) and was restricted to only a few neurons in the adult thoracic ganglion (Fig 2B). To benchmark Insc-GAL4 and Pros-GAL4, two independent pan-neuronal drivers were used, namely *elav-GAL4* and *nSyb-GAL4*. *elav-GAL4* is expressed in all neurons, from newly born to mature, whereas *nSyb-GAL4* expression is confined to mature neurons in which synapse formation has begun, or where synapses have been established. In addition, motor neuron (*D42-GAL4* and *OK371-GAL4*), cholinergic neuron (*Cha-GAL4*), pan-glial (*Repo-GAL4*) and fat body (*CG-GAL4*) *GAL4* drivers were used to manipulate SMN levels. The gross expression patterns of each driver are presented in Fig 2C. The number of larval body wall contractions was again scored when SMN was knocked down using the set of diverse neuronal drivers. As previously described, reduced body wall contraction defects were observed in pan-neuronal *elav-GAL4*; *SMN-RNAi* (Fig 2D), with an approximate 20% reduction in peristatic movements. No contraction defects were observed for nSyb-GAL4 or the motor neuron, cholinergic neuron, glial or fat-body drivers. Contraction defects were observed when SMN-RNAi was expressed in neurogenic cell types. A reduction in contractions was seen with both *Insc-GAL4* and *Pros-GAL4* expressed SMN-RNAi (15% and 14%, respectively (Fig 2D)). In addition, the effect of cell-type-specific *Smn* knockdown on survival to the adult stage was assessed. This was achieved by scoring the number of pupated larvae that emerged as adults (Fig 2E). As previously described, a reduction in eclosion rate was observed in pan-neuronally (*elav-GAL4)* driven SMN-RNAi (Fig 2E), with around 6% fewer flies eclosing in each case, compared with the two control groups. Furthermore, *Insc-GAL4* and *Pros-GAL4* expressed SMN-RNAi also decreased fly eclosion number, with around 7% and 8% less flies hatching, respectively.

**Fig 3:**
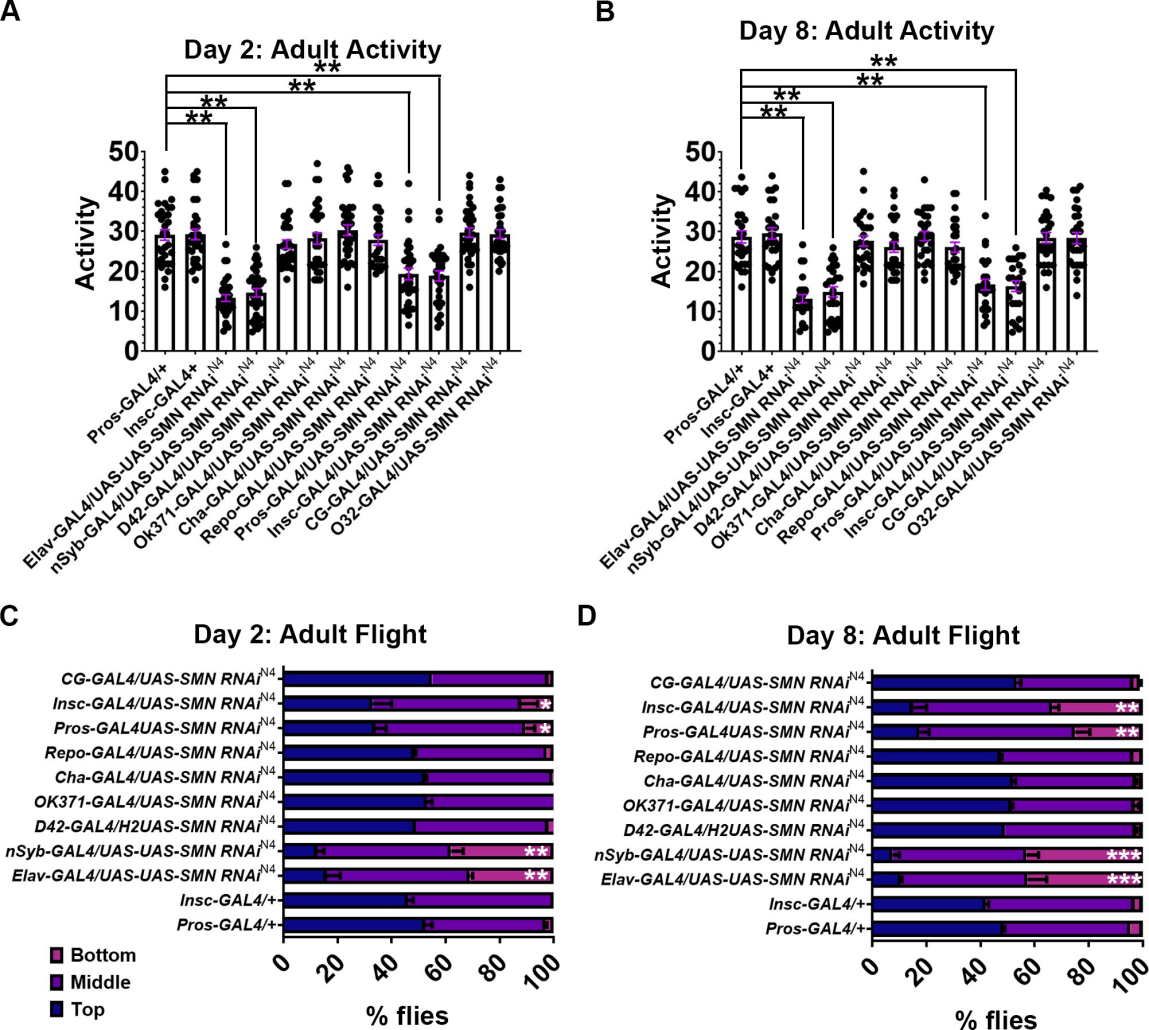
*Survival motor neuron* (*Smn*) knockdown in neurogenic cell types leads to adult motor dysfunction. Flies were tested for motor activity, (A) and (B), and flight ability (C) and (D) at 2 days (A) and (C), and 8 Days (B) and (D). *Drosophila* activity was detected in adult flies over 1 day using the Trikinetics activity monitors in controlled conditions; (A and B) only *Pros-GAL4*/*UAS-SMN-RNAi*^*N4*^ and *Insc-GAL4/UAS-SMN-RNAi*^*N4*^ progressively declined in activity over 2 (F; **P < 0.01, *n* = 20; Kruskal–Wallis test with Dunn’s multiple comparisons) and 8 days (G; ***P < 0.001; *n* = 20; Kruskal–Wallis test with Dunn’s multiple comparisons); (C and D) *Pros-GAL4*; *UAS-SMN-RNAi*^*N4*^ and *Insc-GAL4/UAS-SMN-RNAi*^*N4*^ flies showed a significant reduction in flight ability, with more flies residing at the bottom of the chamber, over 2 (F; *P < 0.05; **P < 0.01; *n* = 40; Kruskal–Wallis test with Dunn’s multiple comparisons) and 8 days (G; ***P < 0.001; *n* = 40; Kruskal–Wallis test with Dunn’s multiple comparisons). All error bars [SEM].

Adult motor defects in the SMN knockdown lines were then assessed. Two phenotypes were selected to assess the effect on locomotion: adult activity and flight performance (Fig 3). *Drosophila* behaviour was analysed using adult flies over two 1-day periods using an environmentally controlled digital activity monitor (Chiu *et al*., 2010) and by flight testing using the Seymour Benzer method [57]. For the full panel of drivers, activity and flight response was scored at 2 and 8 days after eclosion. As previously reported, flight and adult activity defects were observed when *SMN-RNAi^N4^* was expressed using both pan neuronal drivers, *nSyb-GAL4* and *Elav-GAL4*. No significant activity or flight defects, however, were observed when SMN-RNAi^N4^ was expressed in motor neurons, interneurons or sensory neurons at 2 or 8 days. In contrast, *Pros-GAL4* and *Insc-GAL4* driven SMN knockdown displayed a reduction in activity at 2 (Fig 3A) and 8 days (Fig 3B), and a progressive deterioration of flight ability at 2 (Fig 3C) and 8 days (Fig 3D). In summary, SMN reduction in neuronal progenitor cells, and with early to mature pan-neuronal expression, causes both larval and adult locomotor defects.

### Motor defects persist with developmentally targeted spatiotemporal SMN knock down

**Fig 4:**
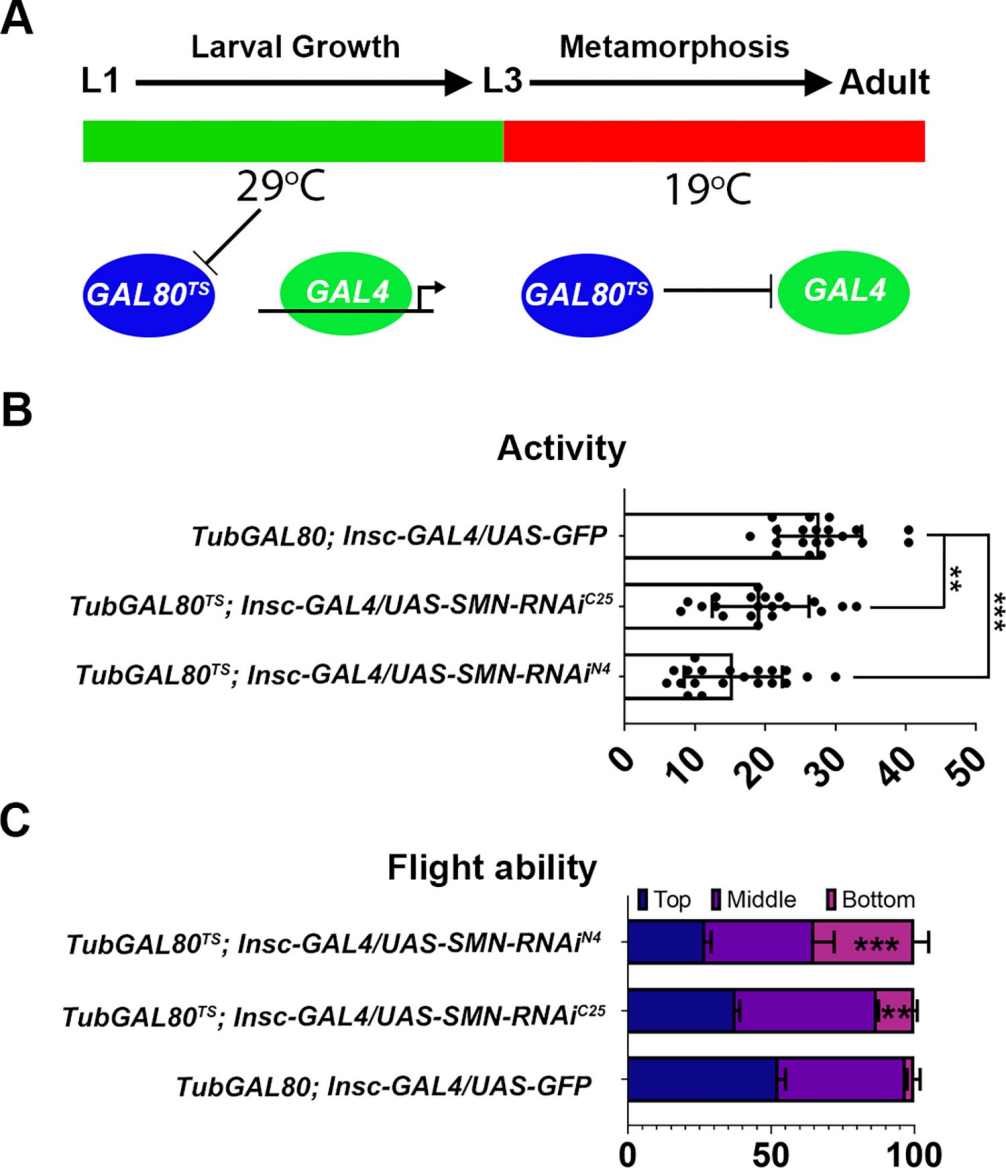
Adult motor defects persist with developmentally targeted spatiotemporal *survival motor neuron* (SMN) knockdown. (A) The GAL80^TS^ system was used to eliminate any adult GAL4 expression. Larvae were reared at 29°C (GAL80^TS^ is inactive; GAL4 is active) and then switched to 19°C (GAL80^TS^ is active; GAL4 is repressed) during pupation; (B and C) two non-overlapping RNAi constructs were used (SMN-RNAi^N4^ and SMN-RNAi^C25^) for flight and activity defects. Both (B) activity (SMN-RNAi^N4^_,_ ***P < 0.001; SMN-RNAi^C25^, **P < 0.01, *n* = 20, Kruskal–Wallis test with Dunn’s multiple comparisons) and (C) flight defects (SMN-RNAi^N4^, ***P < 0.001; SMN-RNAi^C25^, **P < 0.01, *n* = 40; Kruskal–Wallis test with Dunn’s multiple comparisons) were detected using this method.

Although GAL4 drivers generally express in defined cell types, transient or background expression may occur in other tissues or developmental stages. To limit this problem, and confirm the importance of the link between neuroblast and neuronal progenitor specific abnormalities and the motor dysfunction observed in the SMA model, SMN was knocked down with SMN-RNAi constructs using the GAL80^TS^ (TARGET) system [44]. The GAL80^TS^ system further refines transgene expression by targeting spatially confined GAL4 drivers to a specific developmental time period. In addition to the neuroblast-constrained driver (*Insc-GAL4*), the GAL80^TS^ system uses a temperature-sensitive GAL80 transgene (GAL80^TS^) that represses GAL4 at low temperatures (e.g. 19°C), but becomes inactive when the temperature is shifted to 29°C, allowing GAL4 to be expressed [44]. This system was used to initially rear *TubGAL80 and Insc-GAL4/UAS-SMN-RNAi* larvae at 29°C (GAL80^TS^ is inactive; GAL4 and SMN RNAi is expressed). Larvae were then switched to 19°C (GAL80^TS^ is active; the GAL4 gene is repressed; SMN RNAi is not expressed) at the start of pupation, to remove background SMN knockdown in differentiated adult neurons (Fig 3A). qRT-PCR control experiments, using a GFP reporter, highlighted that *Insc-Gal4* did not drive GFP expression after switching to 19°C (S3 Fig). *Drosophila* motor behaviour was again analysed using activity monitoring and flight testing, and an additional SMN RNAi construct was used (SMN-RNAi^C25^). The use of SMN-RNAi^C25^, which behaves as a hypermorph weaker than N4, enabled the comparison of two non-overlapping SMN RNAi constructs that have previously been shown to drive mild phenotypes [39]. Eight days after eclosing, both activity (Fig 4B) (SMN-RNAi^N4^_,_; SMN-RNAi^C25^) and flight defects (Fig 4C) (SMN-RNAi^N4^; SMN-RNAi^C25^) were detected using this method. Therefore, it was confirmed that locomotor defects persist when SMN knockdown using neuroblast drivers is combined with the temporally restricted GAL80^TS^ TARGET system.

### Smn expression in neuroblasts partially rescues survival and motor defects

**Fig 5:**
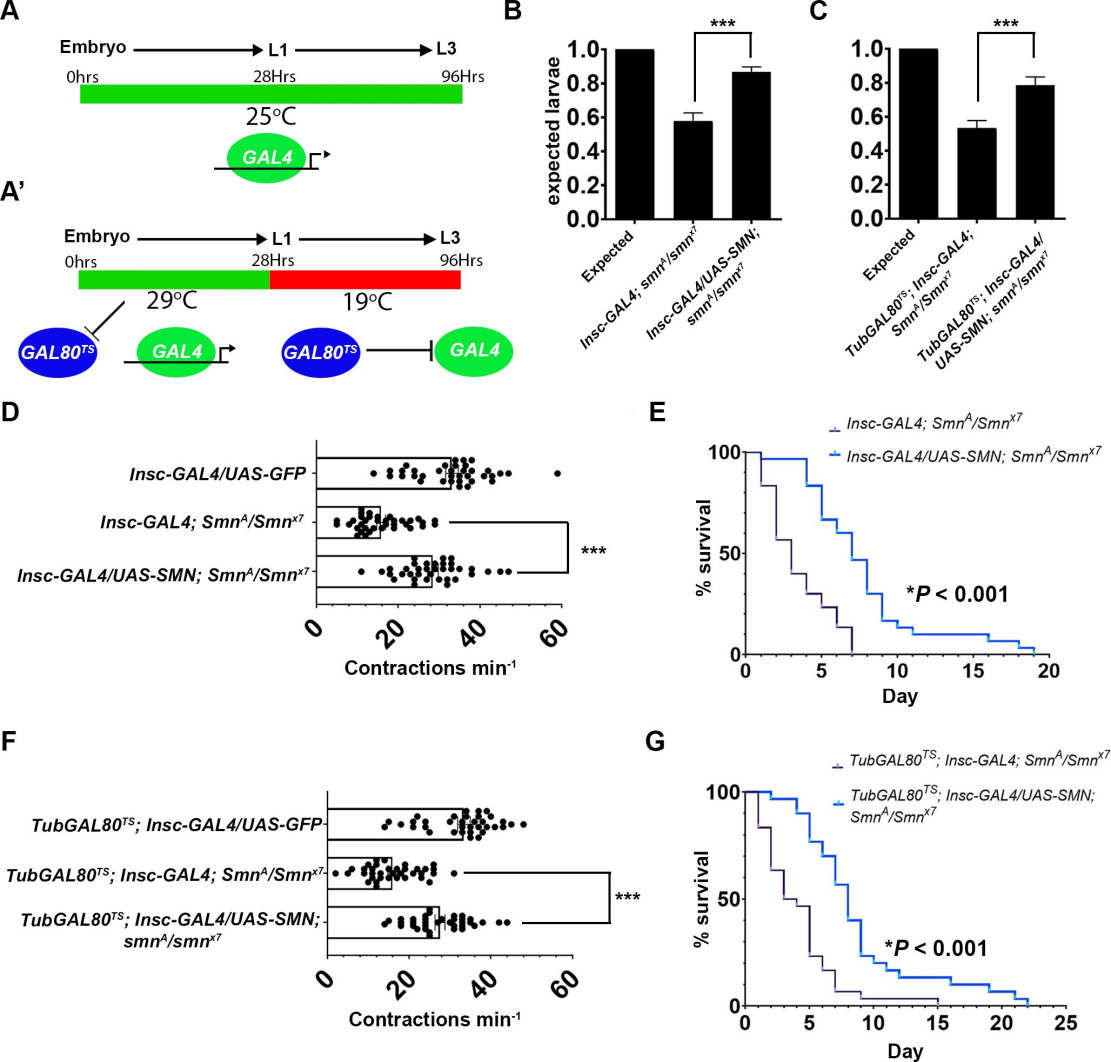
Restoration of *survival motor neuron* (SMN) in neurogenic cell types rescues the motor phenotypes in *Smn* mutant larvae. For rescue studies, both the classical binary GAL4 system (A) and the GAL80^TS^ system (A’) were used. For targeting, a temperature sensitive GAL80 (GAL80^TS^) represses GAL4 at 19°C but becomes inactive at 29°C. Embryos were reared for 24 h at 29°C, during which GAL4 is expressed, then switched to 19°C to eliminate expression. Expression of SMN using Insc-GAL4 rescue the embryonic attrition seen in SMN mutants with both the (B) binary and (C) TARGET *GAL80*^*TS*^ GAL4 systems (***P < 0.001, three experiments for each genotype, each with *n* = 60; Kruskal–Wallis test with Dunn’s multiple comparisons); (D) *Insc-GAL4/UAS-SMN*; *Smn*^*A*^/*S*mn^*x7*^ larvae show significant rescue of locomotor activity at 72 h compared with mutant Insc-GAL4; *Smn*^*A*^/*Smn*^*x7*^ (***P < 0.001, *n* = 15, Kruskal–Wallis test with Dunn’s multiple comparisons); (E) larval survival was extended from a median of 3 days to a median of 7 days (three experiments for each genotype, each with *n* > 30; ***P < 0.001; Mantel-Cox); (F) Tub-GAL80^TS^; *Insc-GAL4/UAS-SMN*; *Smn*^*A*^*/Smn*^*x7*^ larvae display a significant rescue of motor function at 72 h, compared with controls (***P < 0.001, *n* = 20, Kruskal–Wallis test with Dunn’s multiple comparisons); (G) larval survival was extended from a median of 4 days to a median of 8 days (three experiments for each genotype, each with *n* > 30; ***P < 0.001; Mantel-Cox). All error bars [SEM].

Whether expression of SMN in the developing nervous system can rescue the larval locomotor function and survival defects observed in mutant *Smn* flies was next explored (Fig 5). The classic UAS-GAL4 system and the GAL80^TS^ (TARGET) system was used to drive full length SMN protein in the *Smn* mutants [58]. When using the GAL80^TS^ (TARGET) system, embryos were reared at 29°C for the first 24 h, and then switched to 19°C during larval life to restrict SMN expression to a period of embryonic neurogenesis (Fig 5A). qRT-PCR control experiments, using a GFP reporter, highlighted that *Insc-Gal4* did not drive GFP expression after switching to 19°C (S3 Fig).

Neuroblast expression of SMN (Insc-GAL4/UAS-dSMN; *Smn^x7^*/*Smn^A^*) in *Smn* mutant larvae resulted in a marked improvement in embryo hatching defects, with 81% of expected observed compared with 59% observed in mutants alone (*Insu-Gal4*; *Smn^x7^/Smn^A^*). This result was consistent with the GAL80^TS^ (TARGET) system in which SMN replacement led to an improvement from 53% (TubGAL80^TS^, *Insc-GAL4/UAS-dSMN*; *Smn^x7^*/*Smn^A^*) of expected to 79% of expected (TubGAL80^TS^, *Insc-GAL4/UAS-dSMN*; *Smn^x7^*/*Smn^A^*) larval hatching (Fig 5C). In both cases, the compared control was formulated by normalising to the hatching number observed from a *Insc-GAL4/UAS-GFP* cross. Tests were then conducted to see if neurodevelopmental restoration of SMN protein could also rescue larval locomotion and longevity defects. For locomotion, the peristatic contractions in 72 h-old larvae, the time-point at which we see motor defects in *Smn* mutants, were counted. Control larvae (*Insu-Gal4*; *UAS-GFP*), which express GFP in neuroblasts in a wild-type background, underwent an average of 33 contractions per minute (Fig 5D). This contrasted with *Smn* mutant larvae, which underwent an average of 16 peristaltic contractions (Fig 5D) (*Insc-GAL4*; *Smn^x7^/Smn^A^*). When SMN was restored in *Smn* mutant neuroblasts (*Insc-Gal4*, *UAS-SMN*; *Smn^x7^/Smn^A^*), larval movement was significantly rescued, with 28 peristaltic contractions occurring on average (Fig 5D). An assessment of whether neuroblast expression of SMN in the Smn mutant background extended larval life was then made. The *Smn* mutant control larvae (*Insc-GAL4*; *Smn^x7^/Smn^A^*) lived for an average of 3.5 days (Fig 5E). When SMN was restored in *Smn* mutant using the neuroblasts driver (*Insc-Gal4*, *UAS-SMN*; *Smn^x7^/Smn^A^*), average larval life was extended to 7.5 days. These larvae, however, did not pupate, and all died as larvae after 23 days.

To further validate these results, the GAL80^TS^ system was again used to further refine the spatiotemporal window of UAS/GAL4 gene expression (Fig 5A). Smn mutant larvae with targeted *Insc-GAL4* SMN expression (TubGAL80^TS^, *Insc-GAL4/UAS-dSMN*; *Smn^x7^*/*Smn^A^*) displayed significantly rescued peristaltic motor function (27 peristaltic contractions in 1 minute) compared with *Smn* mutants alone (*TubGAL80^TS^*; *Insc-GAL4/+*; *Smn^x7^*/*Smn^A^*), which had averaged 16 peristaltic contractions per minute. The larval longevity analysis was then repeated using the GAL80^TS^ protocol (Fig 5A). When SMN was restored in *Smn* mutants using the neuroblasts driver (TubGAL80^TS^, *Insc-GAL4/UAS-dSMN*; *Smn^x7^*/*Smn^A^*), average larval life was extended to 7.5 days, when compared with *Smn* mutants alone, which only survived on average 4 days (*TubGAL80^TS^*; *Insc-GAL4/+*; *Smn^x7^*/*Smn^A^*). These results show that targeted addition of SMN protein to neuroblast cell populations can partially rescue motor function and longevity defects in the *Drosophila* SMA model.

### Combinatorial immature–mature neuron SMN knockdown synergistically enhances the locomotor and survival phenotypes

**Fig 6:**
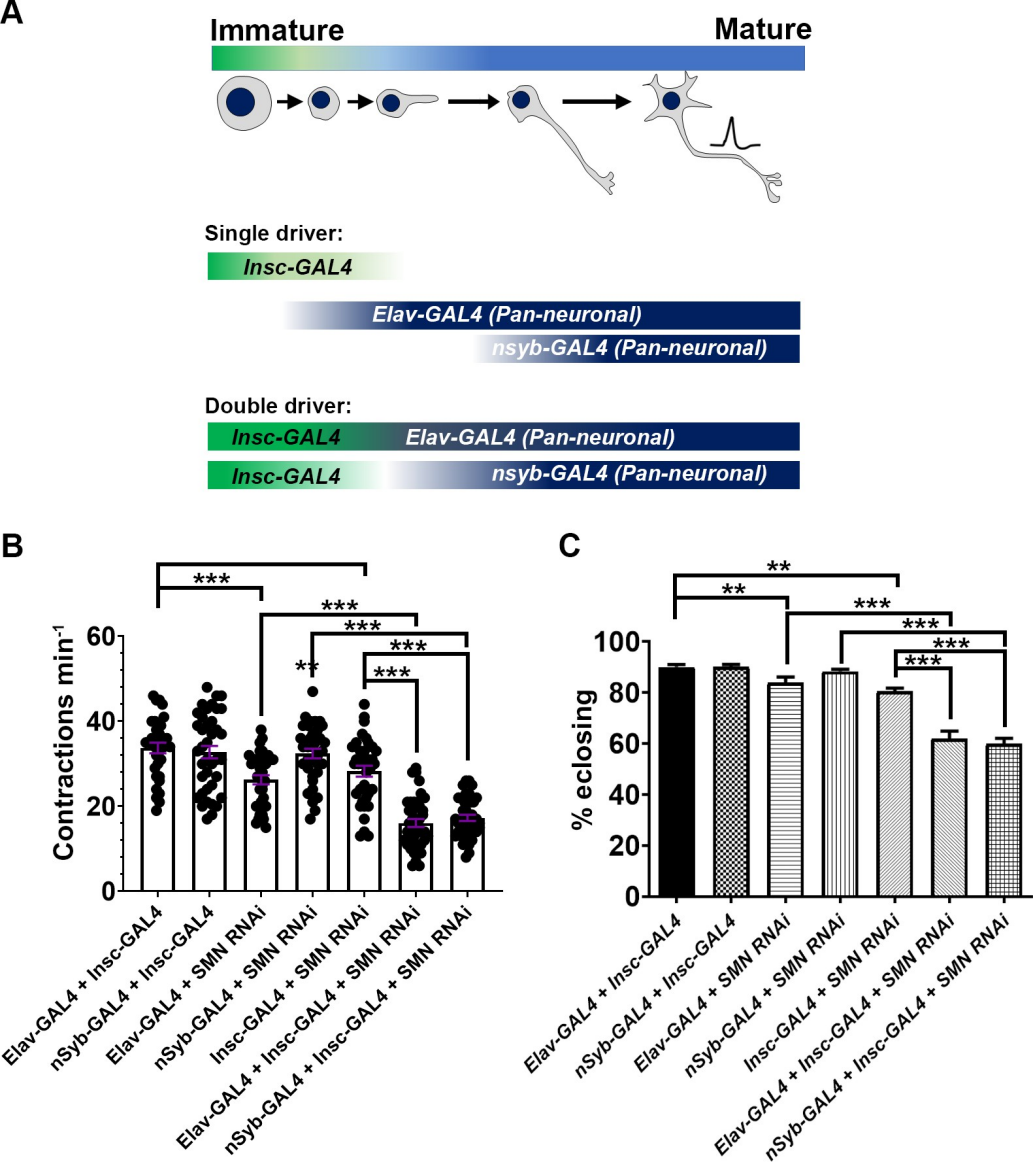
Dual knockdown of *survival motor neuron* (SMN) in neuroblasts and differentiated neurons synergistically enhances the SMA model phenotypes. (A) *GAL4* nervous system expression patterns detailing the driver type and single and double-driver combinations used to knock down *Smn*. For negative controls *Elav-GAL4* + *Insc-GAL4* and *nSyb-GAL4* + *Insc-GAL4* were used. *Elav-GAL4*, *Pros-GAL4* and *Insc-GAL4* driven *UAS-SMN-RNAi*^*N4*^ were used as positive controls and compared with *Elav-GAL4* + *Insc-GAL4* and *nSyb-GAL4* + *Insc-GAL4* driven *UAS-SMN-RNAi*^*N4*^; (B) the genotypes were assessed for locomotor dysfunction at 72 h after hatching. *Elav-GAL4* + *Insc-GAL4* and *nSyb-GAL4* + *Insc-GAL4* driven *UAS-SMN-RNAi*^*N4*^ underwent reduced peristaltic contractions compared with negative and positive controls (***P < 0.001, *n* >20, Kruskal–Wallis test with Dunn’s multiple comparisons); (C) fly hatching number was then assessed to analyse survival to adulthood. *Elav-GAL4* + *Insc-GAL4* and *nSyb-GAL4* + *Insc-GAL4* driven *UAS-SMN-RNAi*^*N4*^ survived compared with negative and positive controls (***P < 0.001; three experiments, each with *n* > 50; Kruskal–Wallis test with Dunn’s multiple comparisons). All error bars [SEM].

Irrespective of the effect of depleting SMN in neuroblasts and their immature daughter cells, loss of SMN in the mature motor system affects survivability and motor function [28,59,60]. The aim was to determine how depletion of SMN in the neuronal progenitor cell types and the mature nervous system enhances the motor and survival phenotypes. Dual SMN RNAi knockdowns were carried out using two combinations: 1) with *Elav-GAL4*, which expresses in both newly born and mature neurons, in conjunction with the neuroblast driver (*Insc-GAL4*); and 2) *nSyb-GAL4*, which expresses in maturing and mature neurons that have undergone synapse formation, in conjunction with the neuroblast driver (*Insc-GAL4*) (Fig 6A). To test these compound knockdowns, larval movement (Fig 6B) and the number of flies that survived to adulthood were analysed (Fig 6C). As previously described, *Elav-GAL4* and *Insc-GAL4* reduced peristaltic contractions, whereas *nSyb-GAL4* SMN knockdown only led to adult movement phenotypes. Controls performed around 33 peristaltic contractions per minute, whereas SMN knockdown independently using Elav-GAL4, nSyb-GAL4 and Insc-GAL4 underwent approximately 26, 32 (non-significant) and 28 peristaltic contractions per minute, respectively (Fig 6B). When Elav-GAL4 and Insc-GAL4 were combined to drive SMN knockdown, larvae underwent 16 peristaltic contractions per minute on average, whereas compound nSyb-GAL4 plus Insc-GAL4 SMN knockdown larvae underwent 17 peristaltic contractions per minute on average (Fig 6B).

A similar trend was seen with eclosion (hatching from the pupal case; Fig 6C). Subtle but significant defects were observed in flies that underwent SMN knockdown with independent *Elav-GAL4* and *Insc-GAL4* expression, with flies hatching at 83% and 80%, respectively, and controls hatching at 90% (*Elav-GAL4* + *Insc-GAL4*, 90%; and *nSyb-GAL4* + *Insc-GAL4*, 90%) (Fig 6C). When *Elav-GAL4* and *Insc-GAL4*, and independently *nSyb-GAL4* and *Insc-GAL4*, were combined to drive SMN knockdown, hatching defects were enhanced (*Elav-GAL4* + *Insc-GAL4* driven *UAS-SMN-RNAi^N^*, 62% hatched; *nSyb-GAL4* + *Insc-GAL4* driven *UAS-SMN-RNAi^N4^*, 60% hatched) (Fig 6C). These data demonstrate that the combinatorial knockdown of SMN in neuroblasts, immature neurons and mature neurons synergistically enhances the motor and survival phenotypes of the *Drosophila* SMA model.

## Discussion

In this study, we show that depleting SMN in neuroblasts and their immature daughter cells can predispose larval and adult *Drosophila* to locomotor dysfunction. In addition, we can partially rescue the larval motor defects of *Smn* mutants by restoring SMN in the neuroblasts and immature developing neurons using targeted expression systems. Finally, we highlight that the combination of presumptive and mature nervous system SMN reduction increases the severity of SMA model phenotypes. We show that the reduction of SMN in cells that are not synapse forming, and thus precede NMJ and sensory-motor network maturation, cause SMA-like phenotypes in the fly.

Although motor neuron loss is also a relatively late feature in SMA patients and mammalian models [25], it is believed that defects in synapse formation and maintenance may be central to the neurological phenotypes observed in SMA patients [59]. Mouse model rescue studies highlight that the therapeutic success of administered rescue constructs generally become progressively diminished only a few days after birth [31–36]. This pre- and peri-natal period coincides with a higher requirement of SMN level in the CNS, a phenomenon also observed in *Drosophila* [5,39,40]. It is difficult to compare the *Drosophila* life cycle with the vertebrate progression of disease; however, the mechanistic and cellular readouts from invertebrate models can offer some degree of translation. Our knockdowns and rescues are limited to neuronal stem cells and their immature progeny. *Drosophila* neuronal stem cells progress through a cascade of transcriptionally distinct identities before permanently differentiating or dying [45,46,61]. During division, this developmental cascade leads to a diversity of developmentally plastic immature daughter cells that undergo further pre- and post-transcriptionally regulated maturation steps, which precede the formation of synapses and ultimately action potentials. Although SMA was classically thought to be a disease of aberrant splicing, the broad requirement for SMN in the regulation of post-transcriptional gene expression is compelling, with roles encompassing snRNP biogenesis [1], mRNP biogenesis [62], mRNA transport [63], ribosomal dynamics [64], chromatin dynamics [65] and translational control [66]. It is probable that deficits in any one of these pathways could lead to stem cell or daughter cell sensitivity to conditions of low SMN. As a stem cell divides and creates a differentiating daughter cell, large changes in alternative splicing drive identity from one of pluripotency to that of an identifiable neuronal lineage with a defined cell biology and physiology [67,68]. It may be that higher SMN levels are required for the temporal–spatial regulation of the alternative splicing events that occur during this switch. Provisional data has shown that both adult flies and larvae display fewer synapses and synaptic boutons respectively, when SMN is knocked down in neuroblasts and the corresponding immature progeny. However, the relevance of bouton number changes has been partly called into question, and these alterations may only be casually linked to the movement defects physiological alterations, and death, observed in *Drosophila* SMA models [49,69]. It may be that upstream functional changes in motor neurons, interneurons, or other neuronal cell types may ultimately lead to the degeneration of the motor neuron or the neuromuscular junction. To this end, the temporal transcription factor cascades that generate the molecular and physiological diversity of the neurons in the developing CNS may be of interest [46, 61]. In future work, we would like to see, when SMN levels are low, if molecular changes in the developing neurons lead to defects in motor neuron physiology, or alterations in the different neuronal classes. We can speculate that changes at this level could alter, in a subtle manner, their molecular identity sensitising neurons to degeneration in certain conditions, or over time.

We have previously reported that SMN overexpression affects developmental timing in *Drosophila* [21] and protects embryonic stem cells from retinol-induced differentiation [19]. In in-vivo SMN mutant neuroblast clones, the levels of both major and minor spliceosome snRNPs (U5 and U2) are reduced in the nucleus of the neuroblast. We have also shown that SMN loss in neuroblasts perturbs cell division and alters the topology of the daughter cell cluster. Furthermore, gene expression analysis conducted on the spinal cord from SMN deficient mice detected changes in proliferative pathways, and identified morphological changes in the dividing cells in the ventral horn [70]. In both mouse and *Drosophila* models, SMN reduction promotes the untimely differentiation of neurons and spermatogonia [19–21], suggesting that high SMN is required for the fidelity of the developmental processes key to cellular differentiation and maturation.

Alternatively, or in-combination, SMN loss may affect downstream translational control. We have previously shown that SMN-deficient neuroblasts display a mislocalisation of a cortical scaffolding protein that binds asymmetrically localised RNP complexes. *Drosophila* neuronal stem cells and neurons alike are energetically demanding, polarised and metabolically distinct [71]; therefore, the presence of highly clustered sites for RNP maturation and processing may be necessary for the correct function of metabolically active and dividing cell types [17]. Nevertheless, it is important to note that cell types and tissue beyond the nervous system are affected by SMN loss [72]. Within our model, although we rescue motor phenotypes and expand lifespan, neuroblast SMN rescue cannot support full larval development, pupariation and pupation to adulthood. In addition, SMN depletion in mature neurons also leads to locomotor dysfunction. Mutations in many widely expressed genes cause selective neuropathies and motor neuron diseases [73]. How we come to think about the nature of these disease genes, particularly their pleiotropy and spatial and temporal requirements, will be key to the eventual revealing of the mechanisms that lead to the selectivity of cell type degeneration.

It seems that, at least in the *Drosophila* model, SMN reduction in many presumptive and mature neuronal cell types can cause locomotor defects and reduce lifespan. To understand the temporal and spatial requirement of SMN during nervous system development, we used *Drosophila* cell and time-specific GAL4 drivers. To date, a large number of UAS/GAL4 studies have investigated how to identify the fly tissue and cell types sensitive of SMN loss [5]. Consistent with other models, ubiquitous SMN knockdown is the most severe, leading to larval lethality, whereas ubiquitous rescue using high level expression drivers confers adult survival [39,40]. Second to this, combinatorial experiments expressing SMN, both early stage pan-neuronal and mesoderm drivers, partially rescues at the adult stage, whereas knockdown using the same driver combination causes larval–pupal lethality [40]. In comparison, pan-neuronal knockdown alone leads to modest adult lethality, neurophysiological and behavioural defects [39,40,74], whereas a subset of other drivers, including those expressed in cholinergic neurons and glutamatergic neurons, have shown specific neuromuscular phenotypes or rescue profiles [41,74]. It is important to highlight that GAL4 drivers will vary in temporal specificity and level. Due to the non-synchronous correlation between transcriptome and proteome [75], enhancer drivers derived from known neuronally expressed genes may generally express earlier or more broadly (at least at lower levels) than expected. These issues highlight the importance of the GAL80^TS^ system used in this study to confine transgene expression to the cell type and time period. However, it is important to note that although the target system offers a high degree of temporal and special control, our study does not fully eliminate the role of low level SMN knockdown and expression in other tissues enhancing the phenotypes and rescues observed, during the period of GAL4 expression.

To summarise, the present research supports that the idea that SMA is caused by a combination of defects that impact motor neuron development, maturation, and maintenance. Moreover, although motor neurons seem to be particularly sensitive to SMN loss, the complex background of multiple tissue defects makes it difficult to unveil the precise timing and nature of the causative defects. The present study directly shows that SMN is required during a window of neurogenesis that precedes synapse formation and neuromuscular junction maturation, and that the motor defects observed in *Drosophila* SMA models can be, in part, be caused by SMN reduction in these cell types. To this end, further study should address how an improper set-up of neuronal networks may compound any motor neuron cell autonomous defects that may arise in SMN-deficient motor neurons.

## Materials and methods

### Drosophila husbandry and stocks

*Smn^x7^* null, *Smn^A^*, *P[UAS-Smn-RNAi]*N4, P[UAS-Smn-RNAi]^C25^ line have been previously described [39, 40]. All stock were backcrossed onto *w^1118^* wild type background. Larvae were grown on apple juice plates with yeast and rich food added. Low population density was maintained for all crosses prior to experimentation. For classical UAS/GAL4 experiments, all crosses were carried out at 25°C to generate extensive but not complete knock-down. GAL4 drivers *1032-GAL*, *D42-GAL4*, *OK371-GAL4*, *Cha-GAL4*, *Repo-GAL4*, and *CG-GAL4* drivers, were obtained from Bloomington (Indiana). *Pros-GAL4* was obtained from the putative-enhancer collection (Bloomington Drosophila Stock Centre [BDSC] at Indiana University, USA). Insu-GAL4; *Tub84B-GAL4^TS^* was a gift from Jürgen Knoblich. Drivers were characterised using *UAS-CD8-GFP*, *UAS-H2B-YFP* (Andrea Brand).

### EdU staining

Dissected CNS were incubated for 1.5 h in 10 μM EdU/Grace’s medium, fixed for 10 min in 4% paraformaldehyde, followed by detection of Alexa Fluor azide according to the Click-iT EdU Imaging Kit (Invitrogen, Waltham, MA, USA) and washing in 0.2% Triton X-100 in phosphate buffered saline. Immunofluorescence was carried out as previously described [21].

### Larval hatching assays

A 2-h lay was carried out on apple juice plates and embryos were lined up in sets of 10. The number of embryos that hatched into larvae was scored for each genotype and was expressed as a percentage of that expected from the lay. *Smn^x7^*/TM6B-GFP and *Smn^A^*/TM6B-GFP were crossed and the number of embryos and larvae with *Smn* heterozygotes) and without GFP expression (homozygous *smn* mutants) were scored as a percentage.

### Larval locomotion assays

Measurement of motor function involved placing individual age-matched third instar larvae at the centre of a 0.7% (weight by volume) agar plate and counting the forward body wall contractions exhibited over 1 min. Larvae were left to acclimatise for 30 s before analysis.

### Larval survival assay

Flies performed a 2hr lay on apple juice plates for 2-h with minimal yeast. Embryos were counted, additional yeast was added, and larval development and death was recorded every 24 h.

### Adult locomotor function assay

Age-matched (1- and 7-day old) male flies were placed individually in a 5-mm glass activity tube containing a food source (5% sucrose [Sigma-Aldrich, St Luis, MO, USA] and 2% Bacto agar [BD Diagnostics, Franklin Lakes, NJ, USA] in distilled water) at one side and a plastic cover with an air hole at the other. The individual glass tubes were placed into the activity monitor (Trikinetics monitors DAM2) (Trikinetics Inc., Waltham, MA, USA) and supported with rubber bands to hold them in place. Locomotor activity was recorded when the flies crossed the infrared light beam at the middle of the glass tubes. Thirty flies were used per genotype and kept under controlled conditions (12-h light–dark cycle at 25°C) for 2 days, day 1 being excluded for habituation. The DAM System collection software was used for collecting data. The raw binary data were processed using DAM Filescan102X (Trikinetics Inc., Waltham, MA, USA) and summed into 1-h bins.

### Adult flight assay

The flight assay was carried out in accordance with a modified protocol originally designed by Benzer [57]. A total of 1000-ml graduated cylinder divided into five sectors was coated internally with mineral oil. Flies were introduced into the top of the cylinder through a funnel and the flies stuck in each sector were counted. The height that flies stick in the cylinder is indicative of their flight capabilities.

### TubGAL80^TS^ TARGET analysis

For larval rescue analysis, GAL80^TS^ analysis, embryos were reared at 29°C (GAL80^TS^ inactive; GAL4 active) and after 24 h (GAL80^TS^ active; GAL4 repressed) and then switched to 19°C during larval life. *TubGAL80^TS^*, *Insc-GAL4/UAS-dSMN*; *Smn^x7^*/*Smn^A^* stock was used and compared with the mutant *TubGAL80^TS^*, *Insc-GAL4/+*; *Smn^x7^*/*Smn^A^* and control *TubGAL80^TS^*, *Insc-GAL4/UAS-GFP* backgrounds. For adult analysis, *Drosophila* larvae were reared at 29°C (GAL80^TS^ inactive; GAL4 active) and then switched to 19°C (GAL80^TS^ active; GAL4 repressed) after pupariation formation. Two non-overlapping RNAi construct was used (SMN-RNAi^N4^ and SMN-RNAi^C25^) and expressed using a *TubGAL80^TS^*, *Insc-GAL4* stock line. *Drosophila* motor behaviour was analysed using activity monitoring, which was carried out at 19°C at days 1 and 7 after hatching. Flight testing was carried out at 8 days.

### qRT-PCR

We determined the levels of GFP mRNA using qPCR methods as described previously [76], using Fast SYBR Green Master Mix (Applied Biosystems Cat. no. 4385612) and the 500 Fast Real-Time PCR System (Applied Biosystems).

### Statistical analysis

A Kruskal–Wallis test and subsequent Dunn’s multiple comparison testing were carried out unless otherwise stated. GraphPad Prism software was used for all data analysis.

## Supporting information

S1 Fig Movement defects present at late larval stages. (A) Control (*w*^*1118*^) and *Smn*^*x7*^/*Smn*^*A*^ larvae were monitored at approximately 24, 48, and 72 ± 1 h after egg laying. Acclimatised larvae were filmed for 1 min, and the distance travelled was traced and measured in cm. *Smn*^*x7*^/*Smn*^*A*^ larvae displayed significant movement defects at 72 h (***P < 0.001, *n* = 20); (B) example superimposed larval locomotion path traces from control and *Smn*^*x7*^/*Smn*^*A*^ mutants for each time point.
(TIF)

S2 Fig Characterisation of Insc-Gal4 expression and targeted SMN knockdown. (A) During larval life, a second wave of larval neuroblast division occurs. The majority of neuroblasts in the ventral ganglion reside at the surface of the larval CNS. (B) Representative *Inscu-GAL4* expression is seen exclusively in neuroblasts and immature neurones in the ventral ganglion and brain lobes. *Insc-Gal4* expression was examined using *UAS-mCherry*. The ventral and medial regions of a third instar larval central nervous system is shown. (C) The larval CNS were co-stained with SMN. The zoom (Box in B) shows SMN staining overlaps with UAS-mCherry immunofluorescence. (D) Expressing *UAS-SMN-RNAi*^*N4*^ exclusively in neuroblasts and immature neurones significantly reduces, but does not eliminate, SMN levels. Edu staining highlights a population of dividing neuroblasts and ganglion mother cells that no longer show SMN enrichment.
(TIF)

S3 Fig Relative expression of GFP mRNA normalised to *rp49* in Tub-GAL80^TS^; *Insc-GAL4/UAS-GFP* during the larval and adults experimental time courses. (A) The GAL80TS system was used to eliminate any adult GAL4 expression. For larval experiments, a temperature sensitive GAL80 (GAL80^TS^) represses GAL4 at 19°C but becomes inactive at 29°C was used. Embryos were reared for 24 h at 29°C, during which GAL4 is expressed, then switched to 19°C to eliminate expression. (B) GFP RNA was measured in whole embryos and larval CNS over the time course analogous to that used in the locomotor and pupation assays. GFP expression was seen to diminish by 0 hrs. We detected no further GFP expression throughout the course of the experimental period. (C) For adult studies, larvae were reared at 29°C (GAL80^TS^ is inactive; GAL4 is active) and then switched to 19°C (GAL80^TS^ is active; GAL4 is repressed) at the start of pupation. (D) Relative expression of GFP mRNA normalised to rp49 in Tub-GAL80^TS^; *Insc-GAL4/UAS-GFP* larvae, pupae and adults. GFP RNA was measured in larval, pupae and adults over the time course analogous to that used in the adult activity and flight assays. The Larvae were switched from 29 to 19°C at the late L3 stage. GFP expression was seen to diminished during larval growth and maturation. We detected no GFP expression throughout the pupal and adult periods studied. (L2, 2^nd^ Instar Larvae; L3, 3rd Instar Larvae).
(TIF)

## References

[1] Liu Q, Fischer U, Wang F, Dreyfuss G. The spinal muscular atrophy disease gene product, SMN, and its associated protein SIP1 are in a complex with spliceosomal snRNP proteins. Cell. 1997;90(6):1013–21. Epub 1997/10/10. doi: 10.1016/s0092-8674(00)80367-0 . 9323129

[2] Lefebvre S, Burglen L, Reboullet S, Clermont O, Burlet P, Viollet L, et al. Identification and characterization of a spinal muscular atrophy-determining gene. Cell. 1995;80(1):155–65. Epub 1995/01/13. doi: 10.1016/0092-8674(95)90460-3 7813012

[3] Chaytow H, Huang YT, Gillingwater TH, Faller KME. The role of survival motor neuron protein (SMN) in protein homeostasis. Cell Mol Life Sci. 2018;75(21):3877–94. Epub 2018/06/07. doi: 10.1007/s00018-018-2849-1 ; PubMed Central PMCID: PMC6182345. 29872871 PMC6182345

[4] Lefebvre S, Burlet P, Liu Q, Bertrandy S, Clermont O, Munnich A, et al. Correlation between severity and SMN protein level in spinal muscular atrophy. Nat Genet. 1997;16(3):265–9. Epub 1997/07/01. doi: 10.1038/ng0797-265 9207792

[5] Grice SJ, Sleigh JN, Liu JL, Sattelle DB. Invertebrate models of spinal muscular atrophy: insights into mechanisms and potential therapeutics. Bioessays. 2011;33(12):956–65. Epub 2011/10/20. doi: 10.1002/bies.201100082 . 22009672

[6] Sleigh JN, Gillingwater TH, Talbot K. The contribution of mouse models to understanding the pathogenesis of spinal muscular atrophy. Dis Model Mech. 2011;4(4):457–67. Epub 2011/06/29. doi: 10.1242/dmm.007245 ; PubMed Central PMCID: PMC3124050. 21708901 PMC3124050

[7] Burghes AH, Beattie CE. Spinal muscular atrophy: why do low levels of survival motor neuron protein make motor neurons sick? Nature reviews Neuroscience. 2009;10(8):597–609. Epub 2009/07/09. doi: 10.1038/nrn2670 ; PubMed Central PMCID: PMC2853768. 19584893 PMC2853768

[8] Fischer U, Liu Q, Dreyfuss G. The SMN-SIP1 complex has an essential role in spliceosomal snRNP biogenesis. Cell. 1997;90(6):1023–9. Epub 1997/10/10. doi: 10.1016/s0092-8674(00)80368-2 9323130

[9] Singh RN, Howell MD, Ottesen EW, Singh NN. Diverse role of survival motor neuron protein. Biochim Biophys Acta Gene Regul Mech. 2017;1860(3):299–315. Epub 2017/01/18. doi: 10.1016/j.bbagrm.2016.12.008 ; PubMed Central PMCID: PMC5325804. 28095296 PMC5325804

[10] Lorson CL, Androphy EJ. An exonic enhancer is required for inclusion of an essential exon in the SMA-determining gene SMN. Human molecular genetics. 2000;9(2):259–65. Epub 1999/12/23. doi: 10.1093/hmg/9.2.259 . 10607836

[11] Monani UR, Lorson CL, Parsons DW, Prior TW, Androphy EJ, Burghes AH, et al. A single nucleotide difference that alters splicing patterns distinguishes the SMA gene SMN1 from the copy gene SMN2. Hum Mol Genet. 1999;8(7):1177–83. Epub 1999/06/17. doi: 10.1093/hmg/8.7.1177 . 10369862

[12] Sleigh JN, Grice SJ, Davies KE, Talbot K. Spinal muscular atrophy at the crossroads of basic science and therapy. Neuromuscul Disord. 2013;23(1):96. Epub 2012/09/18. doi: 10.1016/j.nmd.2012.08.008 . 22981697

[13] Hamilton G, Gillingwater TH. Spinal muscular atrophy: going beyond the motor neuron. Trends Mol Med. 2013;19(1):40–50. Epub 2012/12/12. doi: 10.1016/j.molmed.2012.11.002 . 23228902

[14] Liu Q, Dreyfuss G. A novel nuclear structure containing the survival of motor neurons protein. EMBO J. 1996;15(14):3555–65. Epub 1996/07/15. ; PubMed Central PMCID: PMC451956. 8670859 PMC451956

[15] Jones KW, Gorzynski K, Hales CM, Fischer U, Badbanchi F, Terns RM, et al. Direct interaction of the spinal muscular atrophy disease protein SMN with the small nucleolar RNA-associated protein fibrillarin. J Biol Chem. 2001;276(42):38645–51. Epub 2001/08/18. doi: 10.1074/jbc.M106161200 . 11509571

[16] Liu JL, Gall JG. U bodies are cytoplasmic structures that contain uridine-rich small nuclear ribonucleoproteins and associate with P bodies. Proc Natl Acad Sci U S A. 2007;104(28):11655–9. Epub 2007/06/28. doi: 10.1073/pnas.0704977104 ; PubMed Central PMCID: PMC1899408. 17595295 PMC1899408

[17] Stanek D. Cajal bodies and snRNPs—friends with benefits. RNA Biol. 2017;14(6):671–9. Epub 2016/09/16. doi: 10.1080/15476286.2016.1231359 ; PubMed Central PMCID: PMC5519240. 27627834 PMC5519240

[18] Gabanella F, Carissimi C, Usiello A, Pellizzoni L. The activity of the spinal muscular atrophy protein is regulated during development and cellular differentiation. Hum Mol Genet. 2005;14(23):3629–42. Epub 2005/10/21. doi: 10.1093/hmg/ddi390 . 16236758

[19] Chang WF, Xu J, Chang CC, Yang SH, Li HY, Hsieh-Li HM, et al. SMN is required for the maintenance of embryonic stem cells and neuronal differentiation in mice. Brain Struct Funct. 2015;220(3):1539–53. Epub 2014/03/19. doi: 10.1007/s00429-014-0743-7 . 24633826

[20] Chang WF, Xu J, Lin TY, Hsu J, Hsieh-Li HM, Hwu YM, et al. Survival Motor Neuron Protein Participates in Mouse Germ Cell Development and Spermatogonium Maintenance. Int J Mol Sci. 2020;21(3). Epub 2020/01/30. doi: 10.3390/ijms21030794 ; PubMed Central PMCID: PMC7037566. 31991812 PMC7037566

[21] Grice SJ, Liu JL. Survival motor neuron protein regulates stem cell division, proliferation, and differentiation in Drosophila. PLoS Genet. 2011;7(4):e1002030. Epub 2011/04/15. doi: 10.1371/journal.pgen.1002030 ; PubMed Central PMCID: PMC3072375. 21490958 PMC3072375

[22] Park GH, Maeno-Hikichi Y, Awano T, Landmesser LT, Monani UR. Reduced survival of motor neuron (SMN) protein in motor neuronal progenitors functions cell autonomously to cause spinal muscular atrophy in model mice expressing the human centromeric (SMN2) gene. J Neurosci. 2010;30(36):12005–19. Epub 2010/09/10. doi: 10.1523/JNEUROSCI.2208-10.2010 ; PubMed Central PMCID: PMC2944776. 20826664 PMC2944776

[23] Martinez TL, Kong L, Wang X, Osborne MA, Crowder ME, Van Meerbeke JP, et al. Survival motor neuron protein in motor neurons determines synaptic integrity in spinal muscular atrophy. J Neurosci. 2012;32(25):8703–15. Epub 2012/06/23. doi: 10.1523/JNEUROSCI.0204-12.2012 ; PubMed Central PMCID: PMC3462658. 22723710 PMC3462658

[24] Gogliotti RG, Quinlan KA, Barlow CB, Heier CR, Heckman CJ, Didonato CJ. Motor neuron rescue in spinal muscular atrophy mice demonstrates that sensory-motor defects are a consequence, not a cause, of motor neuron dysfunction. J Neurosci. 2012;32(11):3818–29. Epub 2012/03/17. doi: 10.1523/JNEUROSCI.5775-11.2012 ; PubMed Central PMCID: PMC3679185. 22423102 PMC3679185

[25] Monani UR, Sendtner M, Coovert DD, Parsons DW, Andreassi C, Le TT, et al. The human centromeric survival motor neuron gene (SMN2) rescues embryonic lethality in Smn(-/-) mice and results in a mouse with spinal muscular atrophy. Hum Mol Genet. 2000;9(3):333–9. Epub 2000/02/03. doi: 10.1093/hmg/9.3.333 . 10655541

[26] Lutz CM, Kariya S, Patruni S, Osborne MA, Liu D, Henderson CE, et al. Postsymptomatic restoration of SMN rescues the disease phenotype in a mouse model of severe spinal muscular atrophy. J Clin Invest. 2011;121(8):3029–41. Epub 2011/07/26. doi: 10.1172/JCI57291 ; PubMed Central PMCID: PMC3148744. 21785219 PMC3148744

[27] Martinez-Hernandez R, Bernal S, Also-Rallo E, Alias L, Barcelo MJ, Hereu M, et al. Synaptic defects in type I spinal muscular atrophy in human development. J Pathol. 2013;229(1):49–61. Epub 2012/08/01. doi: 10.1002/path.4080 . 22847626

[28] Le TT, McGovern VL, Alwine IE, Wang X, Massoni-Laporte A, Rich MM, et al. Temporal requirement for high SMN expression in SMA mice. Hum Mol Genet. 2011;20(18):3578–91. Epub 2011/06/16. doi: 10.1093/hmg/ddr275 ; PubMed Central PMCID: PMC3159555. 21672919 PMC3159555

[29] Kariya S, Mauricio R, Dai Y, Monani UR. The neuroprotective factor Wld(s) fails to mitigate distal axonal and neuromuscular junction (NMJ) defects in mouse models of spinal muscular atrophy. Neurosci Lett. 2009;449(3):246–51. Epub 2008/11/18. doi: 10.1016/j.neulet.2008.10.107 ; PubMed Central PMCID: PMC2671206. 19010394 PMC2671206

[30] Dangouloff T, Servais L. Clinical Evidence Supporting Early Treatment Of Patients With Spinal Muscular Atrophy: Current Perspectives. Ther Clin Risk Manag. 2019;15:1153–61. Epub 2019/10/22. doi: 10.2147/TCRM.S172291 ; PubMed Central PMCID: PMC6778729. 31632042 PMC6778729

[31] Foust KD, Wang X, McGovern VL, Braun L, Bevan AK, Haidet AM, et al. Rescue of the spinal muscular atrophy phenotype in a mouse model by early postnatal delivery of SMN. Nat Biotechnol. 2010;28(3):271–4. Epub 2010/03/02. doi: 10.1038/nbt.1610 ; PubMed Central PMCID: PMC2889698. 20190738 PMC2889698

[32] Hua Y, Sahashi K, Hung G, Rigo F, Passini MA, Bennett CF, et al. Antisense correction of SMN2 splicing in the CNS rescues necrosis in a type III SMA mouse model. Genes Dev. 2010;24(15):1634–44. Epub 2010/07/14. doi: 10.1101/gad.1941310 ; PubMed Central PMCID: PMC2912561. 20624852 PMC2912561

[33] Hua Y, Sahashi K, Rigo F, Hung G, Horev G, Bennett CF, et al. Peripheral SMN restoration is essential for long-term rescue of a severe spinal muscular atrophy mouse model. Nature. 2011;478(7367):123–6. Epub 2011/10/08. doi: 10.1038/nature10485 ; PubMed Central PMCID: PMC3191865. 21979052 PMC3191865

[34] Porensky PN, Mitrpant C, McGovern VL, Bevan AK, Foust KD, Kaspar BK, et al. A single administration of morpholino antisense oligomer rescues spinal muscular atrophy in mouse. Hum Mol Genet. 2012;21(7):1625–38. Epub 2011/12/22. doi: 10.1093/hmg/ddr600 ; PubMed Central PMCID: PMC3298284. 22186025 PMC3298284

[35] Bogdanik LP, Osborne MA, Davis C, Martin WP, Austin A, Rigo F, et al. Systemic, postsymptomatic antisense oligonucleotide rescues motor unit maturation delay in a new mouse model for type II/III spinal muscular atrophy. Proc Natl Acad Sci U S A. 2015;112(43):E5863–72. Epub 2015/10/16. doi: 10.1073/pnas.1509758112 ; PubMed Central PMCID: PMC4629342. 26460027 PMC4629342

[36] Zhou H, Meng J, Marrosu E, Janghra N, Morgan J, Muntoni F. Repeated low doses of morpholino antisense oligomer: an intermediate mouse model of spinal muscular atrophy to explore the window of therapeutic response. Hum Mol Genet. 2015;24(22):6265–77. Epub 2015/08/13. doi: 10.1093/hmg/ddv329 ; PubMed Central PMCID: PMC4614699. 26264577 PMC4614699

[37] Tosolini AP, Sleigh JN. Motor Neuron Gene Therapy: Lessons from Spinal Muscular Atrophy for Amyotrophic Lateral Sclerosis. Front Mol Neurosci. 2017;10:405. Epub 2017/12/23. doi: 10.3389/fnmol.2017.00405 ; PubMed Central PMCID: PMC5725447. 29270111 PMC5725447

[38] Lee S, Sayin A, Cauchi RJ, Grice S, Burdett H, Baban D, et al. Genome-wide expression analysis of a spinal muscular atrophy model: towards discovery of new drug targets. PloS one. 2008;3(1):e1404. Epub 2008/01/03. doi: 10.1371/journal.pone.0001404 ; PubMed Central PMCID: PMC2151137. 18167563 PMC2151137

[39] Chang HC, Dimlich DN, Yokokura T, Mukherjee A, Kankel MW, Sen A, et al. Modeling spinal muscular atrophy in Drosophila. PLoS One. 2008;3(9):e3209. Epub 2008/09/16. doi: 10.1371/journal.pone.0003209 ; PubMed Central PMCID: PMC2527655. 18791638 PMC2527655

[40] Chan YB, Miguel-Aliaga I, Franks C, Thomas N, Trulzsch B, Sattelle DB, et al. Neuromuscular defects in a Drosophila survival motor neuron gene mutant. Hum Mol Genet. 2003;12(12):1367–76. Epub 2003/06/05. doi: 10.1093/hmg/ddg157 . 12783845

[41] Imlach WL, Beck ES, Choi BJ, Lotti F, Pellizzoni L, McCabe BD. SMN is required for sensory-motor circuit function in Drosophila. Cell. 2012;151(2):427–39. Epub 2012/10/16. doi: 10.1016/j.cell.2012.09.011 ; PubMed Central PMCID: PMC3475188. 23063130 PMC3475188

[42] McGovern VL, Gavrilina TO, Beattie CE, Burghes AH. Embryonic motor axon development in the severe SMA mouse. Hum Mol Genet. 2008;17(18):2900–9. Epub 2008/07/08. doi: 10.1093/hmg/ddn189 ; PubMed Central PMCID: PMC2722893. 18603534 PMC2722893

[43] Kong L, Valdivia DO, Simon CM, Hassinan CW, Delestree N, Ramos DM, et al. Impaired prenatal motor axon development necessitates early therapeutic intervention in severe SMA. Sci Transl Med. 2021;13(578). Epub 2021/01/29. doi: 10.1126/scitranslmed.abb6871 ; PubMed Central PMCID: PMC8208236. 33504650 PMC8208236

[44] McGuire SE, Mao Z, Davis RL. Spatiotemporal gene expression targeting with the TARGET and gene-switch systems in Drosophila. Science’s STKE: signal transduction knowledge environment. 2004;2004(220):pl6. Epub 2004/02/19. doi: 10.1126/stke.2202004pl6 . 14970377

[45] Egger B, Chell JM, Brand AH. Insights into neural stem cell biology from flies. Philos Trans R Soc Lond B Biol Sci. 2008;363(1489):39–56. Epub 2007/02/21. doi: 10.1098/rstb.2006.2011 ; PubMed Central PMCID: PMC2213715. 17309865 PMC2213715

[46] Homem CC, Knoblich JA. Drosophila neuroblasts: a model for stem cell biology. Development. 2012;139(23):4297–310. Epub 2012/11/08. doi: 10.1242/dev.080515 . 23132240

[47] Sousa-Nunes R, Cheng LY, Gould AP. Regulating neural proliferation in the Drosophila CNS. Curr Opin Neurobiol. 2010;20(1):50–7. Epub 2010/01/19. doi: 10.1016/j.conb.2009.12.005 . 20079625

[48] Truman JW, Bate M. Spatial and temporal patterns of neurogenesis in the central nervous system of Drosophila melanogaster. Dev Biol. 1988;125(1):145–57. Epub 1988/01/01. doi: 10.1016/0012-1606(88)90067-x . 3119399

[49] Lotti F, Imlach WL, Saieva L, Beck ES, Hao le T, Li DK, et al. An SMN-dependent U12 splicing event essential for motor circuit function. Cell. 2012;151(2):440–54. Epub 2012/10/16. doi: 10.1016/j.cell.2012.09.012 ; PubMed Central PMCID: PMC3474596. 23063131 PMC3474596

[50] Grice SJ, Sleigh JN, Motley WW, Liu JL, Burgess RW, Talbot K, et al. Dominant, toxic gain-of-function mutations in gars lead to non-cell autonomous neuropathology. Hum Mol Genet. 2015;24(15):4397–406. Epub 2015/05/15. doi: 10.1093/hmg/ddv176 ; PubMed Central PMCID: PMC4492401. 25972375 PMC4492401

[51] Grice SJ, Sleigh JN, Zameel Cader M. Plexin-Semaphorin Signaling Modifies Neuromuscular Defects in a Drosophila Model of Peripheral Neuropathy. Front Mol Neurosci. 2018;11:55. Epub 2018/03/10. doi: 10.3389/fnmol.2018.00055 ; PubMed Central PMCID: PMC5827687. 29520219 PMC5827687

[52] Clark MQ, Zarin AA, Carreira-Rosario A, Doe CQ. Neural circuits driving larval locomotion in Drosophila. Neural Dev. 2018;13(1):6. Epub 2018/04/21. doi: 10.1186/s13064-018-0103-z ; PubMed Central PMCID: PMC5907184. 29673388 PMC5907184

[53] Berger C, Harzer H, Burkard TR, Steinmann J, van der Horst S, Laurenson AS, et al. FACS purification and transcriptome analysis of drosophila neural stem cells reveals a role for Klumpfuss in self-renewal. Cell Rep. 2012;2(2):407–18. Epub 2012/08/14. doi: 10.1016/j.celrep.2012.07.008 ; PubMed Central PMCID: PMC3828055. 22884370 PMC3828055

[54] Harzer H, Berger C, Conder R, Schmauss G, Knoblich JA. FACS purification of Drosophila larval neuroblasts for next-generation sequencing. Nat Protoc. 2013;8(6):1088–99. Epub 2013/05/11. doi: 10.1038/nprot.2013.062 ; PubMed Central PMCID: PMC3930877. 23660757 PMC3930877

[55] Chu-Lagraff Q, Wright DM, McNeil LK, Doe CQ. The prospero gene encodes a divergent homeodomain protein that controls neuronal identity in Drosophila. Dev Suppl. 1991;Suppl 2:79–85. Epub 1991/01/01. . 1842358

[56] Doe CQ, Chu-LaGraff Q, Wright DM, Scott MP. The prospero gene specifies cell fates in the Drosophila central nervous system. Cell. 1991;65(3):451–64. Epub 1991/05/03. doi: 10.1016/0092-8674(91)90463-9 . 1673362

[57] Benzer S. Genetic dissection of behavior. Sci Am. 1973;229(6):24–37. Epub 1973/12/01. doi: 10.1038/scientificamerican1273-24 . 4202065

[58] Duffy JB. GAL4 system in Drosophila: a fly geneticist’s Swiss army knife. Genesis. 2002;34(1–2):1–15. Epub 2002/09/27. doi: 10.1002/gene.10150 . 12324939

[59] Van Alstyne M, Pellizzoni L. Advances in modeling and treating spinal muscular atrophy. Curr Opin Neurol. 2016;29(5):549–56. Epub 2016/07/30. doi: 10.1097/WCO.0000000000000368 ; PubMed Central PMCID: PMC5074385. 27472505 PMC5074385

[60] Kariya S, Obis T, Garone C, Akay T, Sera F, Iwata S, et al. Requirement of enhanced Survival Motoneuron protein imposed during neuromuscular junction maturation. J Clin Invest. 2014;124(2):785–800. Epub 2014/01/28. doi: 10.1172/JCI72017 ; PubMed Central PMCID: PMC3904626. 24463453 PMC3904626

[61] Isshiki T, Pearson B, Holbrook S, Doe CQ. Drosophila neuroblasts sequentially express transcription factors which specify the temporal identity of their neuronal progeny. Cell. 2001;106(4):511–21. Epub 2001/08/30. doi: 10.1016/s0092-8674(01)00465-2 . 11525736

[62] Donlin-Asp PG, Fallini C, Campos J, Chou CC, Merritt ME, Phan HC, et al. The Survival of Motor Neuron Protein Acts as a Molecular Chaperone for mRNP Assembly. Cell Rep. 2017;18(7):1660–73. Epub 2017/02/16. doi: 10.1016/j.celrep.2017.01.059 ; PubMed Central PMCID: PMC5492976. 28199839 PMC5492976

[63] Pagliardini S, Giavazzi A, Setola V, Lizier C, Di Luca M, DeBiasi S, et al. Subcellular localization and axonal transport of the survival motor neuron (SMN) protein in the developing rat spinal cord. Hum Mol Genet. 2000;9(1):47–56. Epub 1999/12/10. doi: 10.1093/hmg/9.1.47 . 10587577

[64] Bernabo P, Tebaldi T, Groen EJN, Lane FM, Perenthaler E, Mattedi F, et al. In Vivo Translatome Profiling in Spinal Muscular Atrophy Reveals a Role for SMN Protein in Ribosome Biology. Cell Rep. 2017;21(4):953–65. Epub 2017/10/27. doi: 10.1016/j.celrep.2017.10.010 ; PubMed Central PMCID: PMC5668566. 29069603 PMC5668566

[65] Sabra M, Texier P, El Maalouf J, Lomonte P. The Tudor protein survival motor neuron (SMN) is a chromatin-binding protein that interacts with methylated lysine 79 of histone H3. J Cell Sci. 2013;126(Pt 16):3664–77. Epub 2013/06/12. doi: 10.1242/jcs.126003 . 23750013

[66] Sanchez G, Dury AY, Murray LM, Biondi O, Tadesse H, El Fatimy R, et al. A novel function for the survival motoneuron protein as a translational regulator. Hum Mol Genet. 2013;22(4):668–84. Epub 2012/11/09. doi: 10.1093/hmg/dds474 . 23136128

[67] Su CH, D D, Tarn WY. Alternative Splicing in Neurogenesis and Brain Development. Front Mol Biosci. 2018;5:12. Epub 2018/02/28. doi: 10.3389/fmolb.2018.00012 ; PubMed Central PMCID: PMC5816070. 29484299 PMC5816070

[68] Grice SJ, Liu JL, Webber C. Synergistic interactions between Drosophila orthologues of genes spanned by de novo human CNVs support multiple-hit models of autism. PLoS Genet. 2015;11(3):e1004998. Epub 2015/03/31. doi: 10.1371/journal.pgen.1004998 ; PubMed Central PMCID: PMC4376901. 25816101 PMC4376901

[69] Spring AM, Raimer AC, Hamilton CD, Schillinger MJ, Matera AG. Comprehensive Modeling of Spinal Muscular Atrophy in Drosophila melanogaster. Front Mol Neurosci. 2019;12:113. Epub 2019/06/04. doi: 10.3389/fnmol.2019.00113 ; PubMed Central PMCID: PMC6532329. 31156382 PMC6532329

[70] Baumer D, Lee S, Nicholson G, Davies JL, Parkinson NJ, Murray LM, et al. Alternative splicing events are a late feature of pathology in a mouse model of spinal muscular atrophy. PLoS Genet. 2009;5(12):e1000773. Epub 2009/12/19. doi: 10.1371/journal.pgen.1000773 ; PubMed Central PMCID: PMC2787017. 20019802 PMC2787017

[71] Tsogtbaatar E, Landin C, Minter-Dykhouse K, Folmes CDL. Energy Metabolism Regulates Stem Cell Pluripotency. Front Cell Dev Biol. 2020;8:87. Epub 2020/03/18. doi: 10.3389/fcell.2020.00087 ; PubMed Central PMCID: PMC7059177. 32181250 PMC7059177

[72] Nash LA, Burns JK, Chardon JW, Kothary R, Parks RJ. Spinal Muscular Atrophy: More than a Disease of Motor Neurons? Curr Mol Med. 2016;16(9):779–92. Epub 2016/11/30. doi: 10.2174/1566524016666161128113338 . 27894243

[73] Liu EY, Cali CP, Lee EB. RNA metabolism in neurodegenerative disease. Dis Model Mech. 2017;10(5):509–18. Epub 2017/05/05. doi: 10.1242/dmm.028613 ; PubMed Central PMCID: PMC5451173. 28468937 PMC5451173

[74] Timmerman C, Sanyal S. Behavioral and electrophysiological outcomes of tissue-specific Smn knockdown in Drosophila melanogaster. Brain Res. 2012;1489:66–80. Epub 2012/10/30. doi: 10.1016/j.brainres.2012.10.035 ; PubMed Central PMCID: PMC3501589. 23103409 PMC3501589

[75] Casas-Vila N, Bluhm A, Sayols S, Dinges N, Dejung M, Altenhein T, et al. The developmental proteome of Drosophila melanogaster. Genome Res. 2017;27(7):1273–85. Epub 2017/04/07. doi: 10.1101/gr.213694.116 ; PubMed Central PMCID: PMC5495078. 28381612 PMC5495078

[76] Azzam G, Liu JL. Only one isoform of Drosophila melanogaster CTP synthase forms the cytoophidium. PLoS Genet. 2013;9(2):e1003256. Epub 2013/03/06. doi: 10.1371/journal.pgen.1003256 ; PubMed Central PMCID: PMC3573105. 23459760 PMC3573105

