# Supplementary figures and images for: Motor defects in a Drosophila model for spinal muscular atrophy result from SMN depletion during early neurogenesis
Source: PLoS Genet. 2022 Jul 25;18(7):e1010325. doi: 10.1371/journal.pgen.1010325 (PMC9352204; doi:10.1371/journal.pgen.1010325)

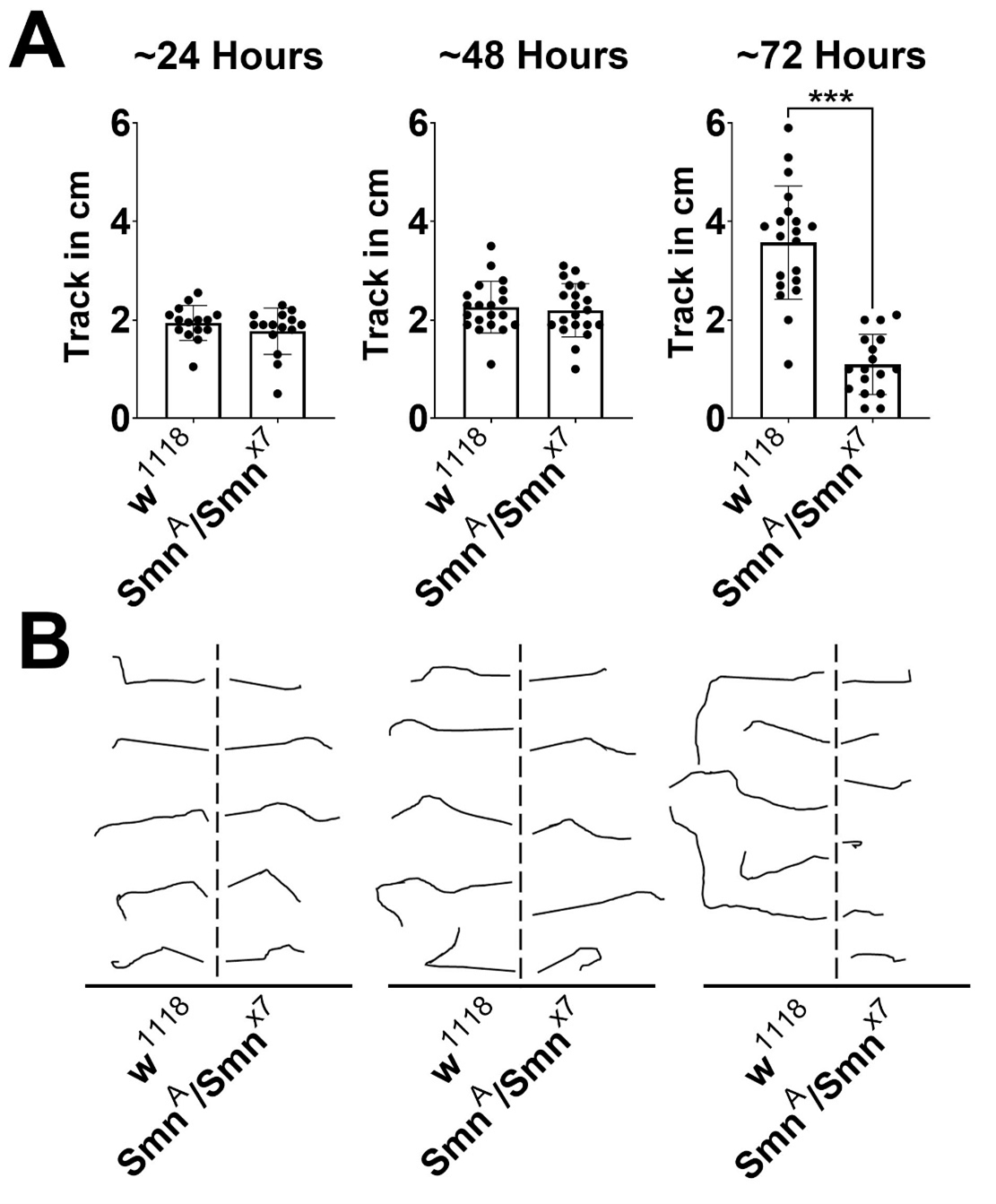

Supplement: S1 Fig — (A) Control (w1118) and Smnx7/SmnA larvae were monitored at approximately 24, 48, and 72 ± 1 h after egg laying. Acclimatised larvae were filmed for 1 min, and the distance travelled was traced and measured in cm. Smnx7/SmnA larvae displayed significant movement defects at 72 h (***P < 0.001, n = 20); (B) example superimposed larval locomotion path traces from control and Smnx7/SmnA mutants for each time point. (TIF) [file pgen.1010325.s001.tif]

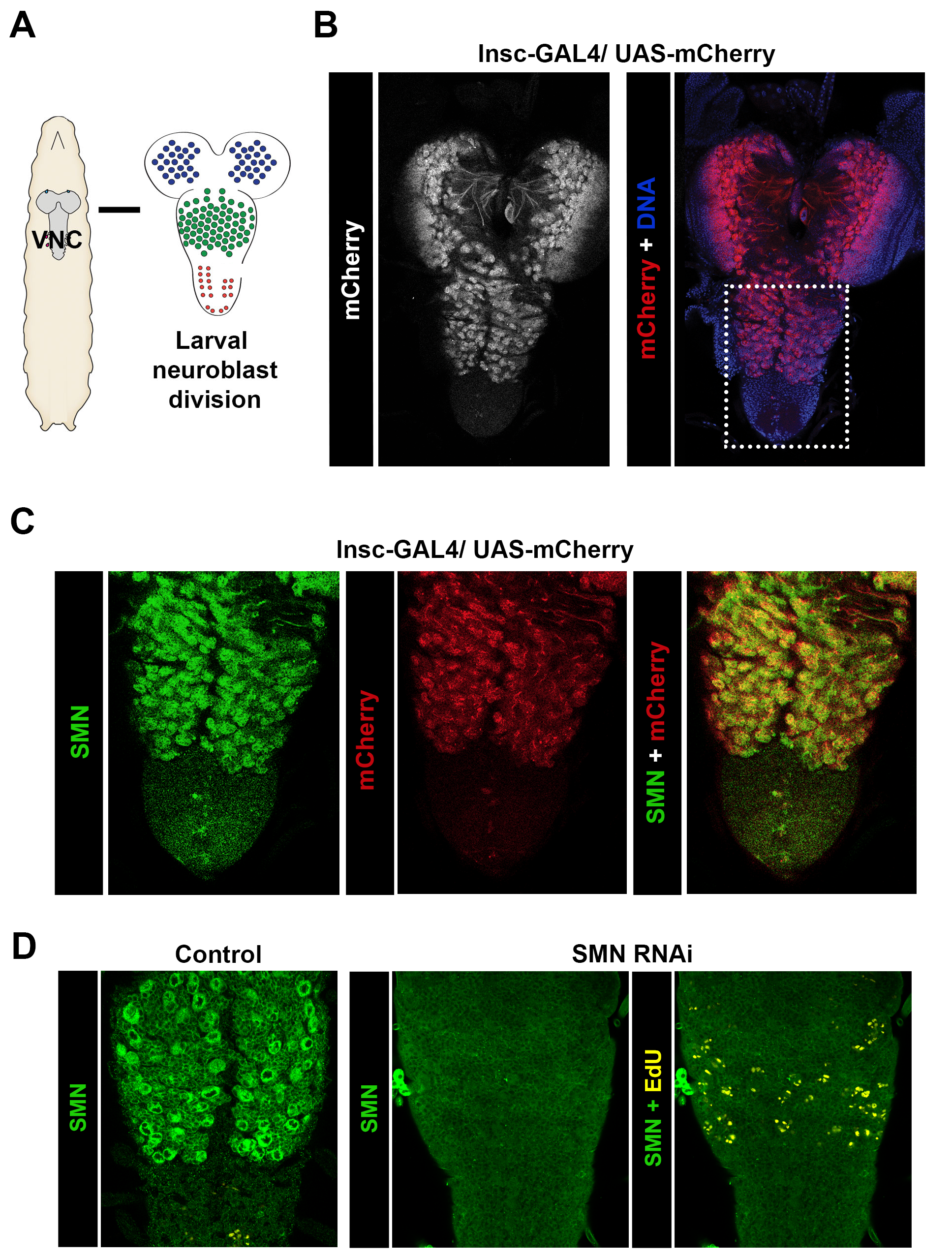

Supplement: S2 Fig — (A) During larval life, a second wave of larval neuroblast division occurs. The majority of neuroblasts in the ventral ganglion reside at the surface of the larval CNS. (B) Representative Inscu-GAL4 expression is seen exclusively in neuroblasts and immature neurones in the ventral ganglion and brain lobes. Insc-Gal4 expression was examined using UAS-mCherry. The ventral and medial regions of a third instar larval central nervous system is shown. (C) The larval CNS were co-stained with SMN. The zoom (Box in B) shows SMN staining overlaps with UAS-mCherry immunofluorescence. (D) Expressing UAS-SMN-RNAiN4 exclusively in neuroblasts and immature neurones significantly reduces, but does not eliminate, SMN levels. Edu staining highlights a population of dividing neuroblasts and ganglion mother cells that no longer show SMN enrichment. (TIF) [file pgen.1010325.s002.tif]

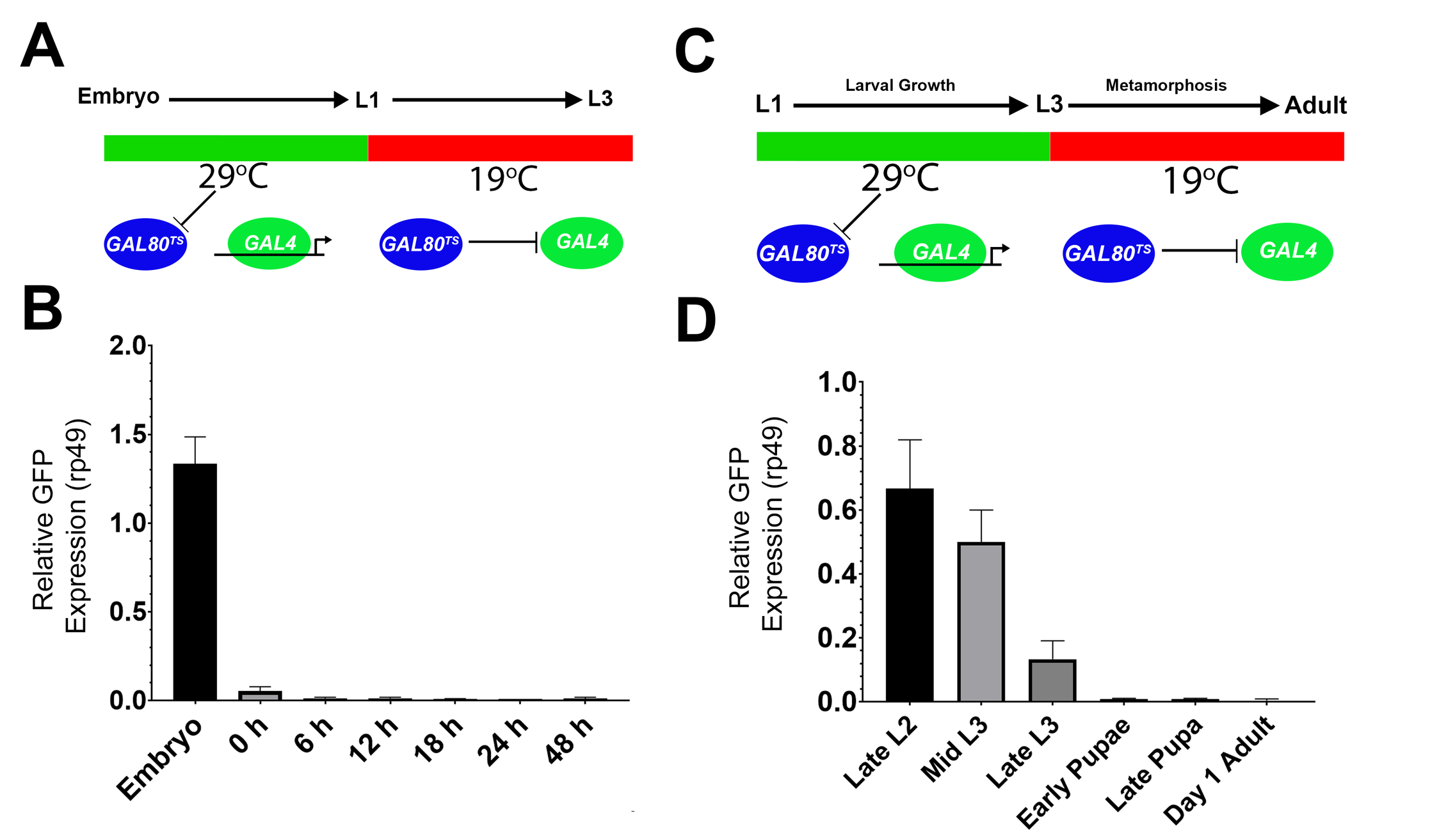

Supplement: S3 Fig — (A) The GAL80TS system was used to eliminate any adult GAL4 expression. For larval experiments, a temperature sensitive GAL80 (GAL80TS) represses GAL4 at 19°C but becomes inactive at 29°C was used. Embryos were reared for 24 h at 29°C, during which GAL4 is expressed, then switched to 19°C to eliminate expression. (B) GFP RNA was measured in whole embryos and larval CNS over the time course analogous to that used in the locomotor and pupation assays. GFP expression was seen to diminish by 0 hrs. We detected no further GFP expression throughout the course of the experimental period. (C) For adult studies, larvae were reared at 29°C (GAL80TS is inactive; GAL4 is active) and then switched to 19°C (GAL80TS is active; GAL4 is repressed) at the start of pupation. (D) Relative expression of GFP mRNA normalised to rp49 in Tub-GAL80TS; Insc-GAL4/UAS-GFP larvae, pupae and adults. GFP RNA was measured in larval, pupae and adults over the time course analogous to that used in the adult activity and flight assays. The Larvae were switched from 29 to 19°C at the late L3 stage. GFP expression was seen to diminished during larval growth and maturation. We detected no GFP expression throughout the pupal and adult periods studied. (L2, 2nd Instar Larvae; L3, 3rd Instar Larvae). (TIF) [file pgen.1010325.s003.tif]
